# De novo transcriptome assembly of the bamboo snout beetle *Cyrtotrachelus buqueti* reveals ability to degrade lignocellulose of bamboo feedstock

**DOI:** 10.1186/s13068-018-1291-9

**Published:** 2018-10-27

**Authors:** Chaobing Luo, Yuanqiu Li, Hong Liao, Yaojun Yang

**Affiliations:** 1 Bamboo Diseases and Pests Control and Resources Development Key Laboratory of Sichuan Province, College of Life Science, Leshan Normal University, No. 778, Riverside Road, Central District, Leshan, 614000 China; 2 College of Food and Biological Engineering, Xihua University, Chengdu, China

**Keywords:** *Cyrtotrachelus buqueti*, Transcriptome, WGCNA, Lignocellulose degradation, Bamboo, CAZyme

## Abstract

**Background:**

The bamboo weevil *Cyrtotrachelus buqueti*, which is considered a pest species, damages bamboo shoots via its piercing–sucking mode of feeding. *C. buqueti* is well known for its ability to transform bamboo shoot biomass into nutrients and energy for growth, development and reproduction with high specificity and efficacy of bioconversion. Woody bamboo is a perennial grass that is a potential feedstock for lignocellulosic biomass because of its high growth rate and lignocellulose content. To verify our hypothesis that *C. buqueti* efficiently degrades bamboo lignocellulose, we assessed the bamboo lignocellulose-degrading ability of this insect through RNA sequencing for identifying a potential route for utilisation of bamboo biomass.

**Results:**

Analysis of carbohydrate-active enzyme (CAZyme) family genes in the developmental transcriptome of *C. buqueti* revealed 1082 unigenes, including 55 glycoside hydrolases (GH) families containing 309 GHs, 51 glycosyltransferases (GT) families containing 329 GTs, 8 carbohydrate esterases (CE) families containing 174 CEs, 6 polysaccharide lyases (PL) families containing 11 PLs, 8 auxiliary activities (AA) families containing 131 enzymes with AAs and 17 carbohydrate-binding modules (CBM) families containing 128 CBMs. We used weighted gene co-expression network analysis to analyse developmental RNA sequencing data, and 19 unique modules were identified in the analysis. Of these modules, the expression of MEyellow module genes was unique and the module included numerous CAZyme family genes. CAZyme genes in this module were divided into two groups depending on whether gene expression was higher in the adult/larval stages or in the egg/pupal stages. Enzyme assays revealed that cellulase activity was highest in the midgut whereas lignin-degrading enzyme activity was highest in the hindgut, consistent with findings from intestinal gene expression studies. We also analysed the expression of CAZyme genes in the transcriptome of *C. buqueti* from two cities and found that several genes were also assigned to CAZyme families. The insect had genes and enzymes associated with lignocellulose degradation, the expression of which differed with developmental stage and intestinal region.

**Conclusion:**

*Cyrtotrachelus buqueti* exhibits lignocellulose degradation-related enzymes and genes, most notably CAZyme family genes. CAZyme family genes showed differences in expression at different developmental stages, with adults being more effective at cellulose degradation and larvae at lignin degradation, as well as at different regions of the intestine, with the midgut being more cellulolytic than the hindgut. This degradative system could be utilised for the bioconversion of bamboo lignocellulosic biomass.

**Electronic supplementary material:**

The online version of this article (10.1186/s13068-018-1291-9) contains supplementary material, which is available to authorized users.

## Background

Lignocellulosic biomass resources are abundant, renewable and environmentally compatible [1]. Therefore, they may become an ideal energy resource for humans. It has been estimated that terrestrial biomass can produce 130 million tonnes of dry wood per year [1–3]. However, the stable structure of lignocellulose leads to a high cost of transformation and processing, which greatly restricts industrialisation. Although lignocellulolytic activity was originally believed to be restricted to plants, bacteria and fungi, evidence has accumulated in recent years for the existence of animal lignocellulolytic enzyme activity (such as cellulases, hemicellulases and lignases), particularly in cellulose-feeding insects [4–7]. These natural biomass utilisation systems (NBUS) are environment-friendly and cost-effective for lignocellulose degradation, and their underlying mechanism could provide the basis for high-efficiency bioconversion of lignocellulose [8].

Of the various NBUS, insects have evolved to efficiently degrade and utilise natural biomass [8, 9]. Such insects are potential candidates for exploring novel lignocellulolytic catalysts because of their diverse and highly adapted lignocellulolytic systems that can efficiently digest a range of lignocellulosic feedstocks [7]. Researchers have reported cellulose digestion in a range of insect species from diverse taxonomic groups [10–13] in more than 10 orders, including Thysanura, Plecoptera, Dictyoptera, Orthoptera, Isoptera, Coleoptera, Trichoptera, Hymenoptera, Phasmida and Diptera [4]. Depending on the insect, the digestibility of lignocellulose reportedly ranges from 11 to 99% [11–13].

Bamboo is considered as a suitable plant for energy utilisation [14–16], and some studies suggest that bamboo is a promising candidate industrial feedstock for lignocellulose biomass because of its high growth efficiency [17–20]. As a relatively recently identified lignocellulose biomass resource, bamboo has attracted increasing interest over the past 5 years as an energy crop [17–24]. However, energy utilisation of bamboo is still in its initial stages, with the main research direction being the cracking of bamboo lignocellulose and the utilisation of bamboo products [25]. Bioconversion offers a new idea for highly efficient conversion of bamboo lignocellulose biomass to fuel ethanol and biodiesel. This challenge now awaits a solution: how to achieve highly efficient bioconversion of bamboo lignocellulose biomass. The bamboo weevil *C. buqueti*, a bamboo plantation pest, causes severe damage to several bamboo species, including *Phyllostachys pubescens*, *Neosinocalamus affinis*, *Bambusa textilis* and *Dendrocalamus farinosus* [26, 27]. This insect damages bamboo shoots via both its piercing–sucking mode of feeding and egg-laying [28]. On the basis of previous research on termites and other beetles that utilise lignocellulosic biomass [29–34], in the present study, we used RNA sequencing and quantification of lignocellulolytic enzyme activity to explore the possibility of bioconversion of lignocellulosic biomass of bamboo feedstock by *C. buqueti*.

## Results

### Prediction of genes encoding carbohydrate-active enzymes in the developmental stage transcriptome of *C. buqueti*

The de novo developmental transcriptome of *C. buqueti* comprised 31,469,916, 36,773,825, 32,128,345, 33,070,448 and 31,434,121 clean reads in eggs, larvae, pupae, female and male imagos, respectively, with a total of 108,854 transcripts obtained and assembled into 83,115 unigenes [35].

The main enzymes related to lignocellulose degradation were CAZymes, which can be divided into the following six categories: glycoside hydrolases (GHs), glycosyltransferases (GTs), polysaccharide lyases (PLs), carbohydrate esterases (CEs), auxiliary activities (AAs) and carbohydrate-binding modules (CBMs) [36]. Consequently, we conducted a search to find all CAZyme genes in the developmental transcriptome. We predicted the total proteins of the *C. buqueti* transcriptome with an *e* value = 1*e*^−5^. The results indicated that 806 unigenes had multiple domains that were assigned to CAZyme families, including 55 GH families 309 GHs, 51 GT families 329 GTs, 8 CE families 174 CEs, 6 PL families 11 PLs, 8 AA families 131 enzymes with AAs and 17 CBM families 128 CBMs (Additional file 1: Table S1; Additional file 2: Table S2). Among these genes, only 99 genes belonged to microbial communities (Additional file 2: Table S2).

The TGH families were primarily represented by GH1, GH9, GH13, GH15, GH16, GH17, GH28, GH38, GH5, GH45, GH47 and GH48 proteins. Of these, 58 belonged to the cellulase group and contained 19 β-glucosidases, 33 endoglucanases and 6 exoglucanases (Table 1). Eleven candidate proteins were identified from the transcriptome as GH1 with β-glucosidase activity predicted by dbCAN CAZyme annotation. Moreover, seven candidate proteins and one protein were identified as GH3 and GH116, respectively, from the *C. buqueti* transcriptome, and also functioned as β-glucosidases. Four GH45 proteins, 23 GH5 proteins, four GH74 proteins and two GH9 proteins were identified as having endoglucanase activity predicted by dbCAN CAZyme annotation. The GH45 class of CAZyme possessed endoglucanase activity and GH9 proteins showed endoglucanase activity, while two GH48 proteins and four GH7 proteins were identified as exhibiting exoglucanase activity.

**Table 1:** Predicted CAZymes and other potential lignocellulolytic enzymes in the *C. buqueti* transcriptome

Enzymes	Specific activity	Locus tag	CAZy modules	e value
Cellulase	β-glucosidase (EC 3.2.1.21)	c65786_g1	GH1	6.40E−150
		c65030_g1	GH1	1.60E−152
		c65030_g6	GH1	1.60E−152
		c97802_g1	GH1	1.60E−152
		c67292_g6	GH1	8.00E−152
		c85745_g1	GH1	8.00E−152
		c64914_g1	GH1	1.60E−144
		c68319_g1	GH1	4.30E−150
		c65934_g1	GH1	5.40E−141
		c68119_g1	GH1	5.40E−141
		c20498_g1	GH116	5.90E−140
		c63009_g1	GH3	3.40E−71
		c43053_g1	GH3	1.50E−70
		c4538_g1	GH3	6.40E−58
		c67417_g1	GH3	9.20E−63
		c88677_g1	GH3	7.90E−64
		c91382_g1	GH3	1.60E−72
		c33332_g1	GH3	2.40E−63
	Endoglucanase (EC 3.2.1.4)	c64192_g1	GH45	5.20E−81
		c57507_g1	GH45	3.40E−83
		c61400_g2	GH45	5.90E−75
		c61400_g1	GH45	1.30E−73
		c65030_g1	GH5	3.00E−10
		c65030_g6	GH5	3.00E−10
		c97802_g1	GH5	3.00E−10
		c60173_g1	GH5	1.80E−06
		c65964_g10	GH5	4.10E−16
		c64914_g1	GH5	2.10E−11
		c54388_g2	GH5	2.00E−15
		c65934_g1	GH5	7.20E−10
		c68119_g1	GH5	7.20E−10
		c40968_g1	GH5	3.10E−09
		c92272_g1	GH5	4.70E−10
		c8325_g1	GH5	2.00E−23
		c60558_g1	GH5	3.80E−33
		c25376_g1	GH5	1.10E−36
		c48026_g1	GH5	1.10E−36
		c49050_g1	GH5	1.10E−36
		c52964_g2	GH5	1.10E−36
		c67425_g1	GH5	1.10E−36
		c90120_g1	GH5	1.10E−36
		c91666_g1	GH5	1.10E−36
		c92778_g1	GH5	1.10E−36
		c39730_g1	GH5	1.10E−31
		c52808_g1	GH5	5.70E−08
		c66553_g1	GH74	2.20E−15
		c69012_g1	GH74	2.50E−16
		c57390_g1	GH74	7.50E−14
		c37145_g1	GH74	7.80E−16
		c53955_g1	GH9	1.60E−131
		c53788_g1	GH9	1.20E−122
	Exoglucanase (EC 3.2.1.176)	c47220_g1	GH48	2.20E−216
		c62602_g1	GH48	2.20E−216
		c45372_g1	GH7	6.50E−193
		c47060_g1	GH7	6.50E−193
		c51841_g1	GH7	5.20E−184
		c69030_g1	GH7	5.20E−184
Hemicellulase	Xylanase (EC 3.2.1.8)	c80201_g1	GH10	1.10E−107
		c80201_g2	GH30	8.80E−08
		c65996_g2	GH30	2.60E−104
		c67805_g2	GH30	2.60E−104
		c40968_g1	GH30	3.00E−116
		c79482_g1	GH30	2.10E−23
Ligninase	Laccase (EC 1.10.3.2)	c65017_g1	AA1	1.00E−64
		c60142_g1	AA1	2.70E−99
		c91035_g1	AA1	1.40E−26
		c53969_g2	AA1	9.30E−109
		c50873_g3	AA1	6.10E−31
		c50873_g1	AA1	2.90E−85
		c64679_g1	AA1	1.50E−104
		c73380_g1	AA1	4.00E−109
		c50873_g2	AA1	1.10E−98
		c64952_g1	AA1	3.10E−99
	Manganese peroxidase (EC 1.11.1.13)	c12756_g1	AA2	2.40E−59
		c63948_g1	AA2	5.60E−35
		c63948_g2	AA2	7.70E−66
		c26986_g1	AA2	3.00E−45
		c63744_g1	AA2	9.30E−62

Six xylanase genes were present in the transcriptome. Numerous genes encoding proteins associated with hemicellulose degradation, such as mannosidase and galactosidase, were also detected (Table 1). Furthermore, 22 β-galactosidases, 25 mannosidases, 17 xyloglucosyltransferases, 82 arylesterases and 75 acetylxylan esterases were identified in the transcriptome (data not shown). The CE10 family exhibited carboxylesterase and xylanase activities as well as mannosidase, galactosidase, xyloglucosyltransferase and acetylxylan esterase activities involved in hemicellulose degradation [37]. These findings indicate that *C. buqueti* has the ability to degrade xylan and other components of hemicellulose.

AAs play an important role in lignin degradation [38]. A total of 131 AA proteins were identified from the developmental transcriptome, including 10 laccases (AA1), 5 MnP (AA2), 46 glucose-methanol-choline (GMC) oxidoreductases (aryl alcohol oxidases and vanillyl-alcohol oxidases; AA3 and AA4, respectively), 4 1,4-benzoquinone reductases (AA6) and other AAs (AA5, AA7 and AA8) (Additional file 1: Table S1). Members of the GMC oxidoreductase superfamily are believed to provide hydrogen peroxide for lignin peroxidase (LiP) and MnP to participate in lignin degradation [39, 40]. The quinone oxidoreductase-derived Fenton chemical reaction reportedly participates in lignocellulose degradation by reducing ions in *Gloeophyllum trabeum* [41].

### Co-expression network analysis of unigenes with weighted gene co-expression network analysis (WGCNA) at different developmental stages

**Fig. 1:**
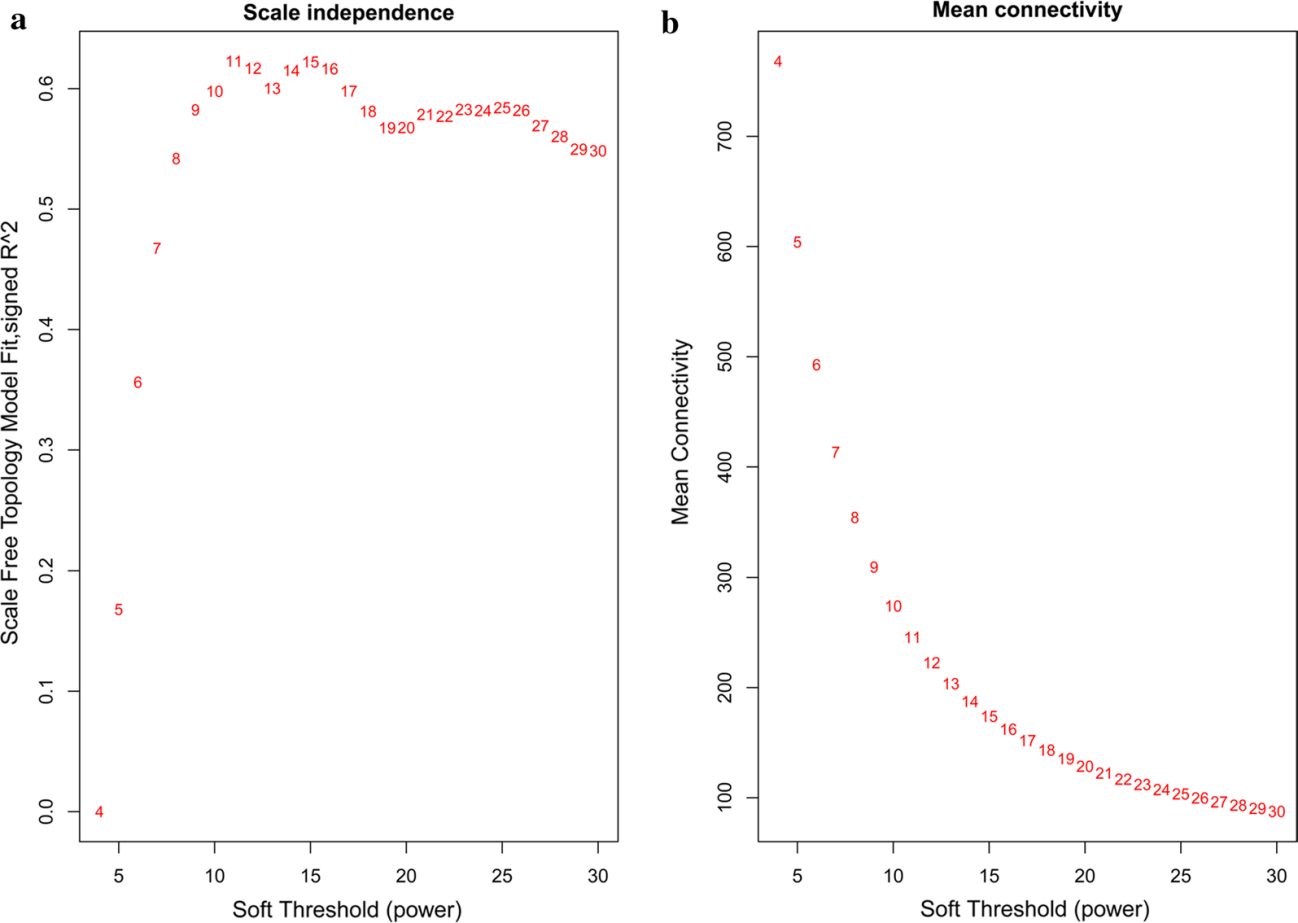
Determination of power beta value based on the adjacency matrix using WGCNA. The adjacency matrix from co-expression data was weighted by the power of correlation data between different genes; i.e. *a*_*ij*_ = |*S*_*ij*_|*β*. The weighted parameter power beta value was determined from the scale-free topology criterion. To ensure that the average connectivity of the network was smooth, we chose *β* = 11 based on both charts: **a** for topology fitting results and **b** for mean connectivity

**Fig. 2:**
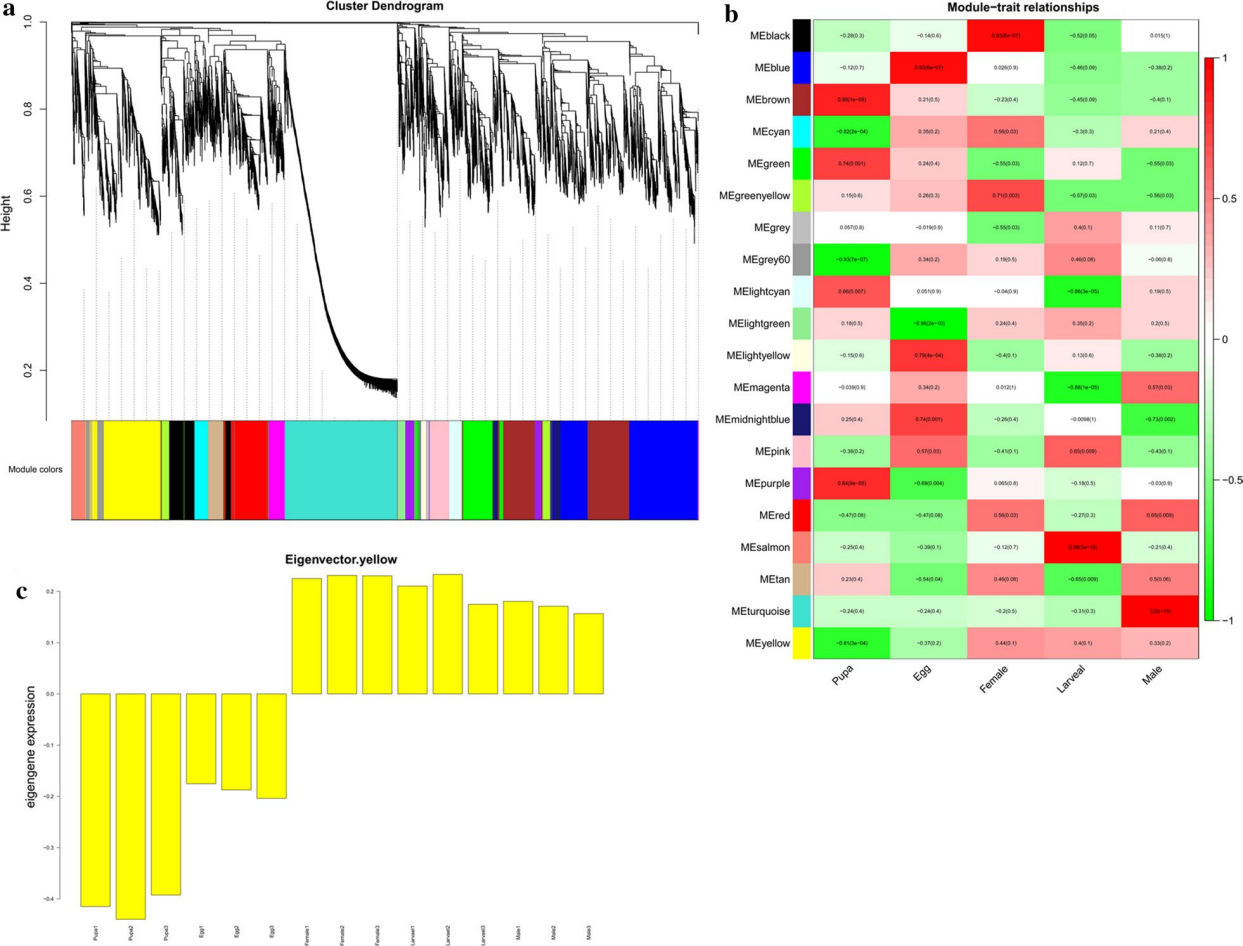
WGCNA analysis of *C. buqueti.* **a** Functional modules are illustrated with different colours. The parameter deepSlip = 4 was set in the WGCNA analysis, which provides high sensitivity to cluster splitting. We additionally required each gene module to contain ≥ 50 genes. In total, 10,789 genes were grouped into 19 modules, which are presented as different colours. The top five modules ordered by the number of genes were turquoise with 1952 genes, blue with 1701 genes, brown with 1333 genes, yellow with 1092 genes and green with 654 genes. The grey colour in the left of the figure represents the seven genes that were not associated with any module. **b** Module-tissue associations. Each row corresponds to a module. Each column corresponds to a specific tissue. The colour of each cell at the row–column intersection indicates the correlation coefficient between the module and developmental stage. A high degree of correlation between a specific module and developmental stage is indicated by dark red or dark green colour. **c** The gene expression patterns in MEyellow module

WGCNA was used for analysing relationships and networks involving the various genes. To build a scale-free network, parameter analysis was performed (Fig. 1). An adjacency function in WGCNA was used to weigh different genes using the following formula: *a*_*ij*_ = (*S*_*ij*_, *β*) = |*S*_*ij*_|^*β*^. As shown in Fig. 1a, we changed the value step-by-step to identify the optimal value, so that the average connectivity of the network was smooth. The value of *β* = 11 was ultimately determined on the basis of the diagnosis chart showing that the average number of co-expressed genes in the final network was 50 (Fig. 1b). As observed in the dendrogram (Fig. 2a), 19 unique module eigengenes were identified (Table 2; Additional file 3: Table S3). Each of the 19 eigengenes correlated with a particular tissue type and developmental stage (Fig. 2b). The three co-expression modules comprised genes that were highly expressed in the egg stage, four in the pupal stage, three in the larval stage, three in female imagos and two in male imagos (*r *> 0.8; Fig. 2b).

**Table 2:** Gene numbers of each module in the WGCNA analysis

Module	Gene numbers
Black	553
Blue	1701
Brown	1333
Cyan	234
Green	654
Green-yellow	274
Grey60	218
Light cyan	222
Light green	130
Light yellow	88
Magenta	283
Midnight blue	233
Pink	390
Purple	274
Red	639
Salmon	242
Tan	270
Turquoise	1952
Yellow	1092

The gene expression patterns of the MEyellow module were divided into the following two types: egg and pupal stages clustered together with a decreasing gene expression level (Fig. 2c); the male, female and larval stages formed another cluster in which gene expression levels increased (Fig. 2c). The egg and pupal stages were dormant and had no obvious foraging activity, while there was a vigorous period of foraging activity in the female imago and larval stages. These findings suggest that the genes in this module may be involved in the life activities of *C. buqueti*, such as foraging.

### Functional enrichment analyses of genes in the MEyellow module

To understand the foraging behaviour of *C. buqueti*, we focussed on the MEyellow module. KEGG pathway and GO enrichment analyses were performed for this model, whereby all genes and hub genes in the MEyellow co-expression module, the first 10% of all genes, were used. According to the GO analysis, all genes in the MEyellow co-expression module were highly enriched in biological processes, such as carbohydrate metabolism, starch metabolism, sucrose metabolism, lipid glycosylation and cellulose catabolism; those enriched in the KEGG pathways were associated with starch and sucrose metabolism, protein digestion and absorption, carbohydrate digestion and absorption, fructose and mannose metabolism and other glycan degradation. Hub genes were mainly enriched in biological processes, such as carbohydrate metabolism, starch metabolism, sucrose metabolism, lipid catabolism, glycogen biosynthesis and cellulose catabolism; those enriched in the KEGG pathways were associated with carbohydrate digestion and absorption and protein digestion and absorption (Table 3).

**Table 3:** The first 20 GO and KEGG items in the WGCNA analysis of the developmental transcriptome

ID	Biological process	Count	ID	KEGG pathway	Count
GO:0005975	Carbohydrate metabolic process	53	ko00040	Pentose and glucuronate interconversions	22
GO:0005982	Starch metabolic process	16	ko00052	Galactose metabolism	19
GO:0005985	Sucrose metabolic process	16	ko04142	Lysosome	29
GO:0030259	Lipid glycosylation	7	ko00983	Drug metabolism—other enzymes	13
GO:0050790	Regulation of catalytic activity	4	ko00500	Starch and sucrose metabolism	18
GO:0006885	Regulation of pH	4	ko04974	Protein digestion and absorption	10
GO:0019530	Taurine metabolic process	4	ko04973	Carbohydrate digestion and absorption	8
GO:0015858	Nucleoside transport	4	ko04080	Neuroactive ligand–receptor interaction	12
GO:0006879	Cellular iron ion homeostasis	4	ko00980	Metabolism of xenobiotics by cytochrome P450	10
GO:0008272	Sulphate transport	4	ko00140	Steroid hormone biosynthesis	6
GO:0006687	Glycosphingolipid metabolic process	5	ko00053	Ascorbate and aldarate metabolism	8
GO:0022900	Electron transport chain	5	ko00511	Other glycan degradation	6
GO:0006771	Riboflavin metabolic process	7	ko00982	Drug metabolism—cytochrome P450	8
GO:0006213	Pyrimidine nucleoside metabolic process	2	ko04612	Antigen processing and presentation	9
GO:0046439	l-Cysteine metabolic process	2	ko04976	Bile secretion	10
GO:0006826	Iron ion transport	3	ko00051	Fructose and mannose metabolism	8
GO:0019497	Hexachlorocyclohexane metabolic process	6	ko00561	Glycerolipid metabolism	11
GO:0030245	Cellulose catabolic process	2	ko00600	Sphingolipid metabolism	6
GO:0034220	Ion transmembrane transport	2	ko00190	Oxidative phosphorylation	19
GO:0016117	Carotenoid biosynthetic process	6	ko00260	Glycine, serine and threonine metabolism	10

**Fig. 3:**
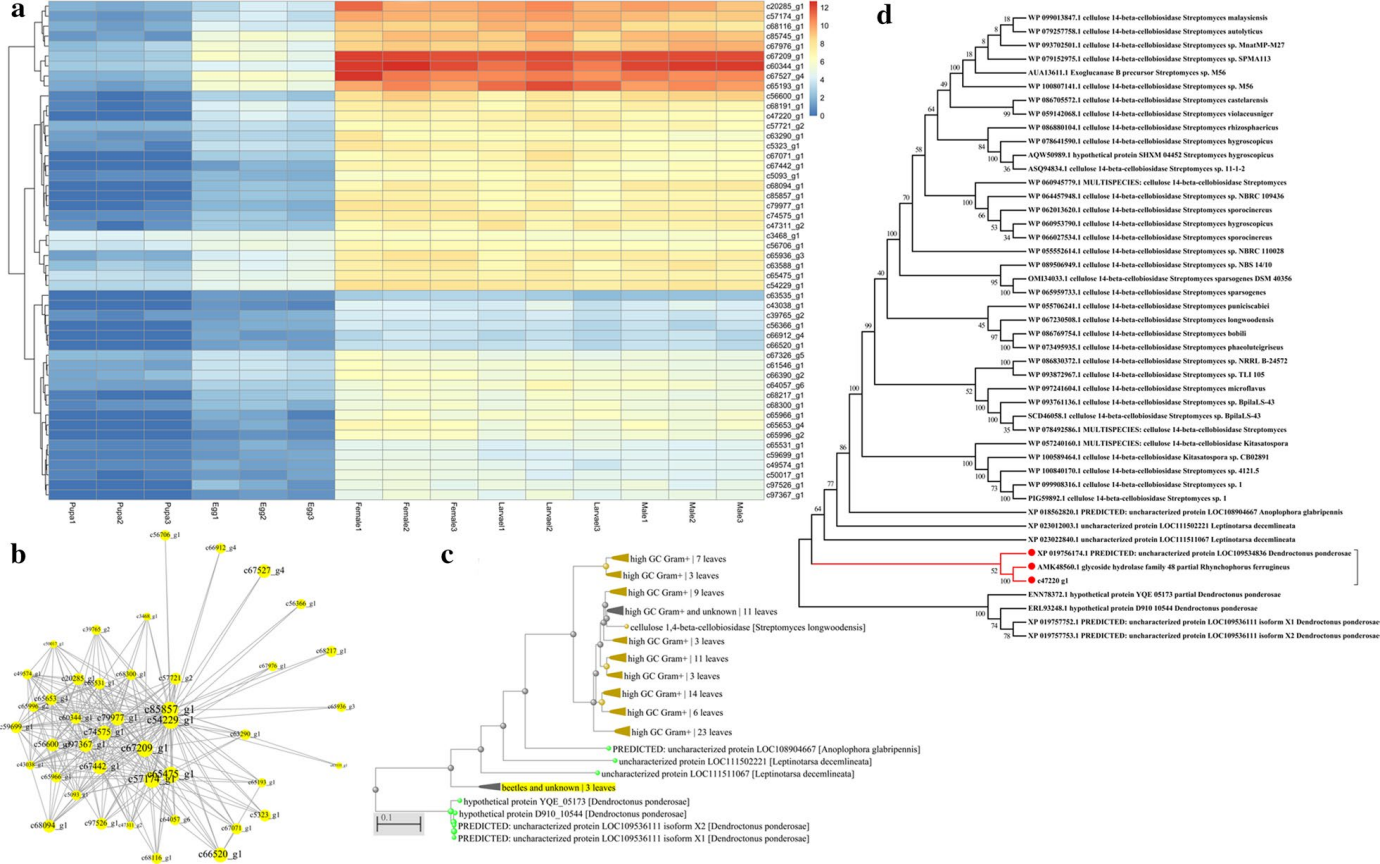
Analysis of genes in the MEyellow module. **a** The heat map of the most highly connected 50 hub genes in the MEyellow module. **b** Co-expression networks of the most highly connected 50 hub genes in the MEyellow module. **c**, **d** Phylogenetic tree of c47220_g1 gene

The 50 most highly connected hub genes in the MEyellow co-expression module were used for analysing gene expression and co-expression networks. Gene expression showed that the expression level in imagos and larval stages was higher than that in egg and pupal stages (Fig. 3a). Co-expression networks showed two core hub genes, namely c85857_g1 and c54229_g1 (Fig. 3b). The c54229_g1 gene belongs to the tetraspanin family, whereas the c85857_g1 gene is of unknown function. Remarkably, in this module, the hub gene c47220_g1 was annotated to the glycoside hydrolase 48 gene family (GH48), which is an important glycoside hydrolase. The GH48 gene family also encodes cellulose exonuclease, which degrades cellulose by the formation of a multi-enzyme cellulosome complex with other glycoside hydrolases or free enzyme systems.

*Cqcbh5* belongs to GH48 and encodes a cellulose exonuclease that acts to degrade cellulose. Phylogenetic analysis revealed that *Cqcbh5* was closely related to the exoglucanase of four phytophagous insects *Dendroctonus ponderosae*, *Rhynchophorus ferrugineus*, *Leptinotarsa decemlineata* and *Anoplophora glabripennis,* as well as to that from some cellulolytic bacteria (Fig. 3c, d). This finding suggests that *Cqcbh5* has a function similar to that of insect and bacterial exoglucanases, which is involved in cellulose degradation. Moreover, the mRNA level of *Cqcbh5* was higher in imago and larval stages than in egg and pupal stages (Fig. 3a), suggesting that the insect can utilise the cellulose of bamboo shoots during these developmental stages.

### Expression of CAZyme family genes in sub-modules

**Fig. 4:**
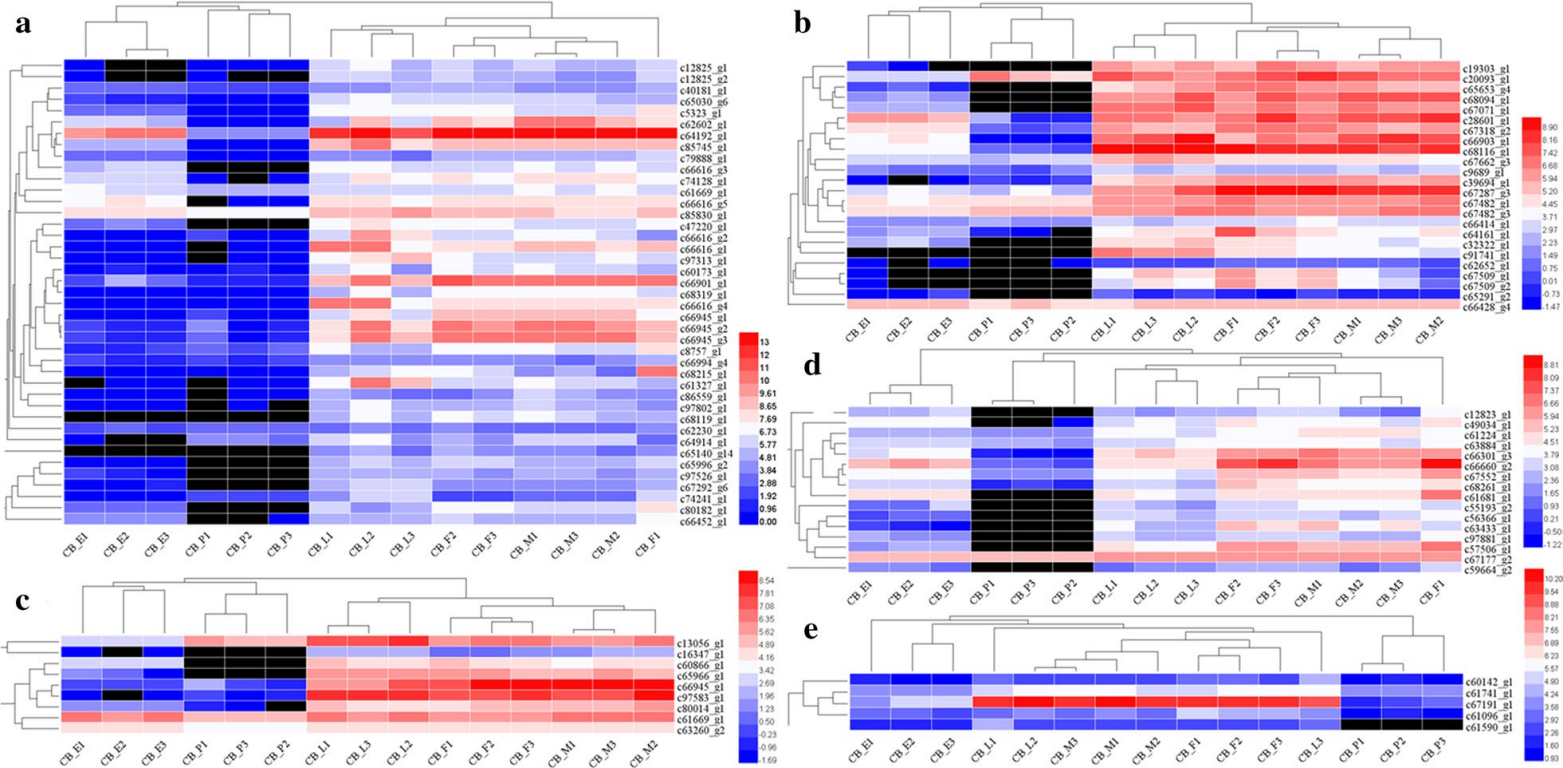
Hierarchical clustering of the expression of CAZyme family genes in the MEyellow module. **a** GHs, **b** GTs, **c** CEs, **d** CBMs and **e** AAs. *GHs* glycoside hydrolases, *GTs* glycosyltransferases, *CEs* carbohydrate esterases, *CBMs* carbohydrate-binding domains, *PLs* polysaccharide lyases, *AAs* auxiliary activities and *CAZymes* carbohydrate-active enzymes. The black area represents an expression level of zero

We screened all CAZyme family genes in the MEyellow module. The MEyellow module contained 41 GHs, 16 GTs, 9 CBMs, 24 CEs and five AAs, whereas PLs were absent. A reads per kilobase per million reads expression heat map for each family of CAZymes in the MEyellow module was generated according to gene expression during development. The expression patterns of these CAZyme family genes were divided into two categories: one for eggs and pupae and another for female and male larvae. Expression levels of CAZyme family genes in adult and larval stages were higher than those in egg and pupal stages in the MEyellow module (Fig. 4a–e). Lignocellulose degradation is mainly associated with the action of proteins encoded by CAZyme family genes [49]. In this study, many CAZyme family genes exhibited higher expression levels in adult and larval stages than in egg and pupal stages. Via its piercing–sucking mode of feeding, *C. buqueti* mainly eats bamboo shoots, which are enriched in carbohydrates, sugars and lignocellulose (Additional file 4: Table S4). These findings indicate that larvae and adults have the ability to convert lignocellulose in bamboo shoots into nutrients and energy for growth.

In the presented summaries of expression patterns of all CAZyme genes in the transcriptome, genes that were not expressed in most samples have been deleted. As shown in Fig. 4S, 391 genes, namely 103 GHs, 132 GTs, 73 CBMs, 55 CEs and 28 AAs, remained for analysis. The expression patterns of GH can be divided into two main categories: one with no obvious differences between the developmental stages and one in which expression is higher in adult and larval stages than in pupal and egg stages (Additional file 5: Fig. S1a). The expression patterns of GT can be grouped into three categories: one with no obvious differences across development, one in which expression is higher in adult and larval stages than in pupal and egg stages and a third in which the expression pattern differs from category two (Additional file 5: Fig. S1b). The expression pattern of CBM was similar to that of GT (Additional file 5: Fig. S1c), whereas the expression pattern of CE was similar to that of GH (Additional file 5: Fig. S1d). AA gene expression did not show significant differences across developmental stages (Additional file 5: Fig. S1e).

### Changes in the expression of carbohydrate metabolism, fatty acid metabolism, protein metabolism and energy metabolism genes in the developmental transcriptome

**Fig. 5:**
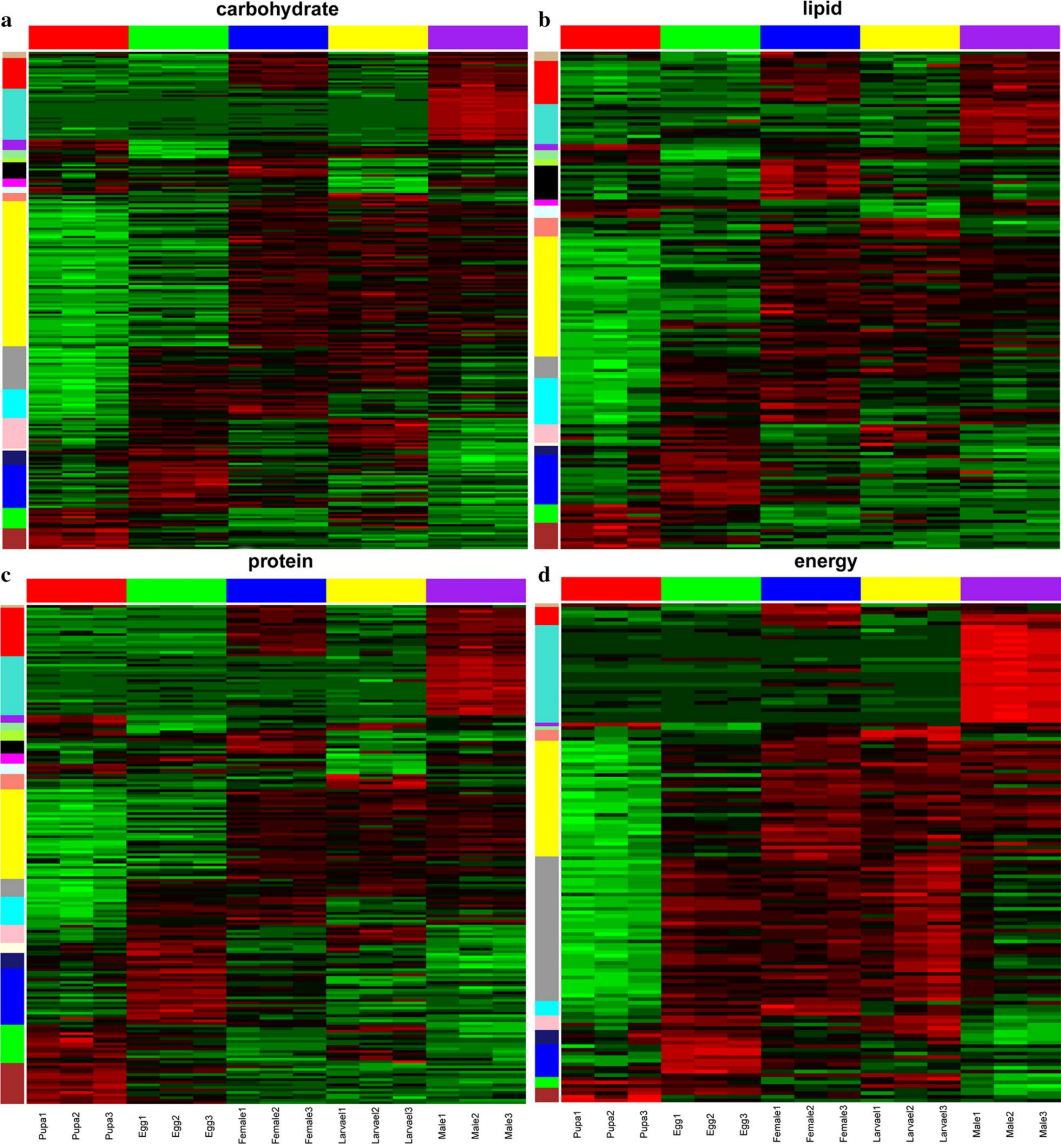
Hierarchical clustering of gene expression involved in nutrient metabolism. **a** Carbohydrate metabolism, **b** lipid metabolism, **c** protein metabolism and **d** energy metabolism

Bamboo shoots are rich in various nutrients (Additional file 4: Table S4), containing abundant carbohydrates, sugars, fats and proteins. It is not clear whether *C. buqueti* can utilise these nutrients or whether their energy metabolism changes after feeding on bamboo shoots. To determine whether *C. buqueti* can efficiently utilise bamboo shoot biomass, we analysed the expression patterns of genes associated with the metabolism of carbohydrates, fatty acids, proteins and energy in the developmental transcriptome. We also assessed whether the expression changed across development and whether any such changes agreed with the feeding habits of the insect. The expression levels of most genes involved in these pathways in the MEyellow co-expression module were higher in imago and larval stages than in egg and pupal stages (Fig. 5a–d). These findings indicate that metabolic pathways operate at a higher rate in adults and larvae and might relate to the ability of adults and larvae to digest carbohydrate, lipids and proteins from bamboo shoots.

### Prediction of carbohydrate-active enzyme gene expression in the imago transcriptome

**Fig. 6:**
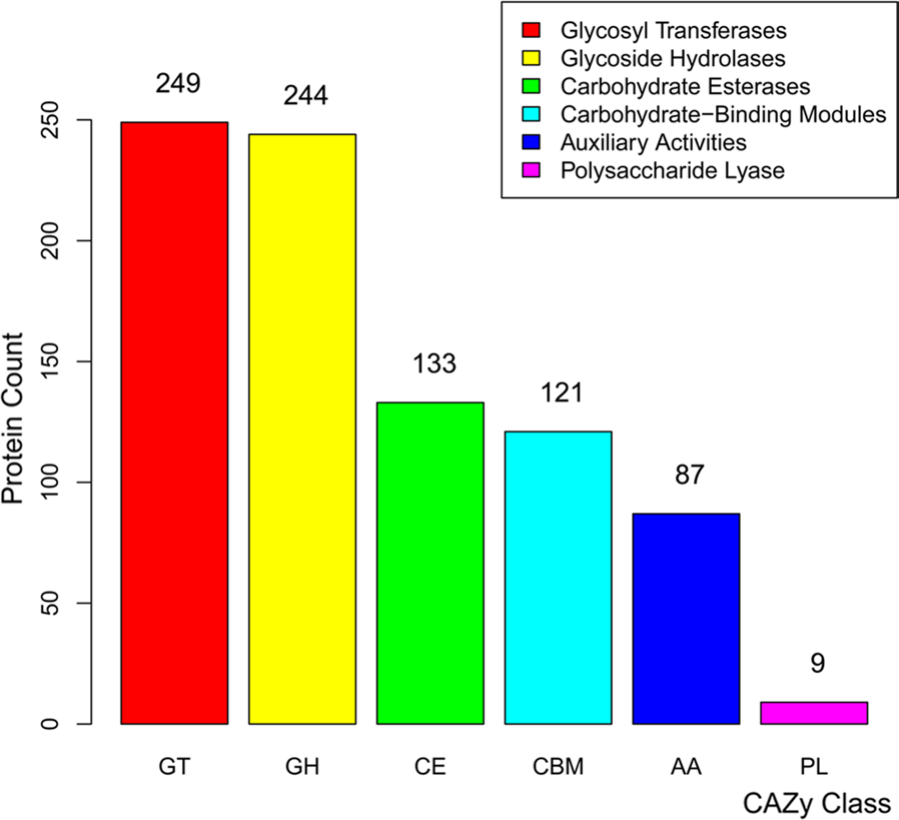
The number of CAZyme genes defined in the transcriptome of Lesham and Chishui. *GHs* glycoside hydrolases, *GTs* glycosyltransferases, *CEs* carbohydrate esterases, *CBMs* carbohydrate-binding domains, *PLs* polysaccharide lyases, *AAs* auxiliary activities and *CAZyme* carbohydrate-active enzymes

In a previous study, we conducted RNA sequencing of the digestive system, reproductive system and muscle tissue of imagos collected in the cities of Leshan and Chishui [42]. There are clear differences in the *C. buqueti* population sizes between the two cities [43]. Our analysis of genes related to lignocellulose degradation in the transcriptomes of these two populations demonstrated that 843 genes had multiple domains assigned to CAZyme families, namely 249 GHs, 244 GTs, 133 CEs, 9 PLs, 87 enzymes with AAs and 121 CBMs; 106 of these proteins also contained signal peptides that were predicted to be extracellular proteins (Fig. 6).

In the transcriptomes of *C. buqueti* in Muchuan and Chishui, there were 19 cellulase genes [including 4 endoglucanase (GH8) genes], 4 exoglucanase (GH10) genes and 11 β-glucosidase genes (GH1, 3) (Table 4). Among the cellulases, seven protein sequences, including endoglucanases, β-glucosidases and exoglucanases, exhibited potential secretion signals. However, there were only three GHs containing CBM domains, which were surprisingly unrelated to cellulose degradation. Three endoglucanase genes (*Cqeng1,* c20964_g1_i1; *Cqeng2*, c31184_g1_i1 and *Cqeng3*, c63642_g1_i1), four exoglucanase genes (*Cqcbh1*, c23242_g1_i1; *Cqcbh2*, c29519_g1_i1; *Cqcbh3*, c49080_g1_i2 and *Cqcbh4*, c49080_g1_i1) and seven β-glucosidase genes (*Cqbgln1*, c31266_g1_i2; *gln2*, c31266_g2_i1; *Cqbgln3*, c31266_g2_i2; *Cqbgln4*, c31732_g6_i1; *Cqbgln5*, c31732_g6_i2; *Cqbgln6*, 31852_g1_i5 and *Cqbgln7*, c31852_g1_i2) were used in a phylogenetic analysis including termites and other beetles to assess the evolutionary relationships with these species (Additional file 6: Fig. S2a–c).

**Table 4:** Predicted CAZymes and other potential lignocellulolytic enzymes in adult transcriptome

Enzymes	Specific activity	Locus tag	CAZy modules
Cellulase	Endoglucanase (EC 3.2.1.4)	c31184_g1_i1	GH8
		c20964_g1_i1	GH8
		c20964_g1_i2	GH8
		c63642_g1_i1	GH8
	Exoglucanase (EC 3.2.1.176)	c23242_g1_i1	GH10
		c49080_g1_i1	GH10
		c49080_g1_i1	GH10
		c29519_g1_i1	GH10
	β-glucosidase (EC 3.2.1.21)	c42084_g1_i1	GH1
		c31266_g1_i1	GH1
		c47702_g1_i1	GH1
		c31732_g6_i1	GH1
		c31732_g6_i2	GH3
		c31266_g1_i2	GH3
		c62209_g1_i1	GH3
		c31266_g2_i2	GH3
		c31266_g2_i1	GH3
		c31852_g1_i1	GH3
		c11294_g1_i1	GH3
Hemicellulase	Xylanase	c68284_g1_i1	GH30
Ligninase	Laccase	c57050_g1_i1	AA1
		c27827_g1_i1	AA1
		c28149_g1_i1	AA1
		c34749_g1_i1	AA1
		c27827_g2_i2	AA1
		c27827_g2_i1	AA1
		c71661_g1_i1	AA1

Although there was only one typical xylanase gene in the *C. buqueti* transcriptome, we found 96 esterase (CE1, 3, 10, 16) genes (Fig. 6), 26 of which possessed signal peptide sequences. Furthermore, some GHs involved in hemicellulosic polysaccharide hydrolysis, such as ɑ-*N*-arabinofuranosidase (GH43, 51), ɑ-mannosidase (GH38) and galactosidase (GH2, 4), have been found in the *C. buqueti* transcriptome.

Many genes encoding enzymes potentially involved in lignin degradation were identified in the *C. buqueti* transcriptome (Fig. 6). Among them, genes for two laccases *Cqlac1* (c27827_g1_i1) and *Cqlac2* (c28149_g1_i1) were used in the phylogenetic analysis. The analysis revealed that *Cqlac1* and *Cqlac2* were closely related to the laccase (Lac) genes of *Monochamus alternatus* and *D. ponderosae* (Additional file 6: Fig. S2c), indicating that insects from different geographical areas exhibit many CAZyme family genes.

### Comparison of enzyme activities at different developmental stages and in different intestinal tissues from imagos or larvae

We detected the activity of several lignocellulolytic enzymes in imagos and larvae. Cellulase activities differed across the various developmental stages, with each enzyme exhibiting different activity patterns. Activity of exoglucanase (CBH), which reached 584.753 ± 91.215 U/g in the foregut of adult females, was higher than that of β-glucosidase (CB) and endoglucanase (EG) in adult females, which was 27.639 ± 9.401 U/g in the hindgut and 235.814 ± 59.925 U/g in the midgut, respectively. In adult males, EG exhibited the highest enzyme activity and CB exhibited the lowest. The enzyme activity pattern in larvae was similar to that in adult males (Table 5). Furthermore, enzyme activity differed between various regions of the intestine. The overall highest CBH activity was observed in the midgut (with the exception of highest activity in the female foregut), whereas the highest CB activity was in the hindgut—particularly the male hindgut (214.597 ± 54.711 U/g). EG activity was highest in the midgut, peaking in males (1744.8271 ± 50.604 U/g). In summary, these results showed that cellulase activities differed according to both developmental stage and intestinal region. These findings suggest that the different aspects of cellulose degradation in *C. buqueti* are performed at different developmental stages and in different parts of the intestine.

**Table 5:** Cellulase activities of *C. buqueti* in different tissues

	Tissues	Female	Male	Larvae	Substrates
Endoglucanase	Mouthpart	167.574 ± 56.143**cA**	36.944 ± 7.116**bB**	22.227 ± 2.293**aB**	Carboxy methyl cellulose (CMC)
	Foregut	584.753 ± 91.215**aA**	168.065 ± 27.839**aB**	0**aB**	
	Midgut	547.303 ± 55.846**aA**	198.834 ± 35.686**aB**	228.197 ± 49.354**aB**	
	Hindgut	119.618 ± 46.375**bcA**	74.403 ± 10.015**aA**	0**aB**	
	Total intestine	333.488 ± 56.600**abcA**	150.491 ± 25.473**aA**	0**aA**	
	Mouthpart + total intestine	237.788 ± 49.687**bcA**	122.701 ± 41.611**aA**	17.936 ± 4.6191**aB**	
Exoglucanase	Mouthpart	0**aA**	54.314 ± 12.635**aA**	30.288 ± 6.118**aA**	Microcrystalline celluose (MC)
	Foregut	0**aB**	15.939 ± 3.643**bA**	44.996 ± 4.774**aA**	
	Midgut	5.021 ± 1.112**aA**	76.401 ± 17.143**aA**	84.693 ± 12.384**aA**	
	Hindgut	27.639 ± 9.401**aA**	214.597 ± 54.711**bA**	0**aB**	
	Total intestine	0**aA**	37.614 ± 4.551**bA**	0**aA**	
	Mouthpart + total intestine	6.768 ± 2.796**aA**	93.996 ± 19.370**bA**	2.821 ± 1.155**aA**	
β-glucosidase	Mouthpart	34.231 ± 10.168**bB**	185.758 ± 53.732**bA**	7.575 ± 2.613**aB**	Salicin
	Foregut	29.294 ± 11.822**bB**	239.177 ± 100.275**bA**	0.4454 ± 0.107**aB**	
	Midgut	235.814 ± 59.925**aB**	1744.827 ± 50.604**aA**	6.487 ± 1.058**aB**	
	Hindgut	22.959 ± 7.533**bA**	0**bA**	0.4217 ± 0.111**aA**	
	Total intestine	53.002 ± 17.693**bA**	382.107 ± 124.953**bA**	2.429 ± 0.669**aA**	
	Mouthpart + total intestine	36.031 ± 10.066**bA**	326.273 ± 117.143**bA**	5.788 ± 0.447**aA**	
Laccase	Mouthpart	0.049 ± 0.001**bB**	0.168 ± 0.003**aA**	0.053 ± 0.003**bB**	2,2,-Azino-bis (3-ethylbenzothiazoline-6-sulfonic acid) (ABTS)
	Foregut	0.004 ± 0.001**bA**	0.192 ± 0.042**aA**	0.029 ± 0.001**bA**	
	Midgut	0.111 ± 0.011**bB**	0.372 ± 0.061**aB**	5.101 ± 0.532a**A**	
	Hindgut	0.065 ± 0.001**bA**	0.113 ± 0.014**aA**	0.003 ± 0.001**bA**	
	Total intestine	0.738 ± 0.231**aA**	0.376 ± 0.072**aA**	0.699 ± 0.099**bA**	
	Mouthpart + total intestine	0.121 ± 0.038**bB**	0.256 ± 0.008**aA**	0.061 ± 0.004**bB**	
Manganese peroxidase	Mouthpart	0.327 ± 0.068**aA**	0.917 ± 0.228**aA**	0.326 ± 0.037**bA**	2,6-Dimethyl phenol (2,6-DMP)
	Foregut	0.322 ± 0.025**aA**	0.235 ± 0.006**aA**	0.057 ± 0.003**bA**	
	Midgut	0.124 ± 0.027**aB**	0.475 ± 0.069**aB**	2.162 ± 0.453a**A**	
	Hindgut	0.558 ± 0.1129**aB**	1.372 ± 0.191**aA**	0.068 ± 0.002a**bA**	
	Total intestine	0.152 ± 0.010**aA**	0.737±0.171**aA**	0.893 ± 0.195**bA**	
	Mouthpart + total intestine	0.082 ± 0.016**aC**	0.324 ± 0.027**aB**	0.772 ± 0.023a**bA**	
Lignin peroxidase	Mouthpart	0.146 ± 0.022**bA**	0.459 ± 0.130**bA**	0.013 ± 0.003**bcB**	Veratryl alcohol (VA)
	Foregut	0.013 ± 0.001**bA**	0**cA**	0.002 ± 0.001**bcA**	
	Midgut	0.437 ± 0.061**bA**	0.471 ± 0.034**bcA**	0.043 ± 0.012**aB**	
	Hindgut	0.051 ± 0.010**abB**	1.453 ± 0.289**aA**	0.008 ± 0.002**cB**	
	Total intestine	0**bA**	0.065 ± 0.014**bcA**	0.026 ± 0.008**bA**	
	Mouthpart + total intestine	0**bB**	0.067 ± 0.016**bcA**	0.012 ± 0.003**bcA**	

A total of 165 female imagos, 165 male imagos and 165 larvae were sampled to determine enzyme activity. Five biological replicates were conducted for each treatment. Descriptive data are expressed as mean ± SEM. Different lowercase letters in the same column indicate significant differences in the different intestinal sections at the 0.05 level (*n* = 5); Different capital letters in the same line indicate significant differences at different developmental stage at the 0.05 level (*n* = 5)

Lignin-degrading enzyme activity also differed according to the developmental stage and intestinal region. Laccase (Lac) activity was highest in the midgut, reaching 5.101 ± 1.171 U/g in larval midgut, which was notably higher than in the adult midgut of adults or other intestinal regions. Manganese peroxidase (MnP) activity was highest in males, by adult females and larvae, whereas whole-intestine enzyme activity was highest in larvae (0.893 ± 0.428 U/g). Among the different parts of the gut, MnP activity was highest in the hindgut of adult females (0.558 ± 0.257 U/g) and males (1.372 ± 0.421 U/g) and in the midgut of larvae (2.162 ± 0.997 U/g). Lignin peroxidase (LiP) activity was highest in the hindgut of males (1.453 ± 0.636 U/g). These results indicate that both adults and larvae of *C. buqueti* have the ability to degrade lignin and that this ability differs according to the developmental stage and intestinal region.

Descriptive data were expressed as mean ± SEM; The different normal letters in the same column indicated significant difference in different intestinal sections at 0.05 level (*n *= 5); The different capital letters in the same line indicated significant difference at different developmental stage at 0.05 level (*n *= 5).

### Expression analysis of lignocellulase genes in different intestine regions in imagos and larvae

**Fig. 7:**
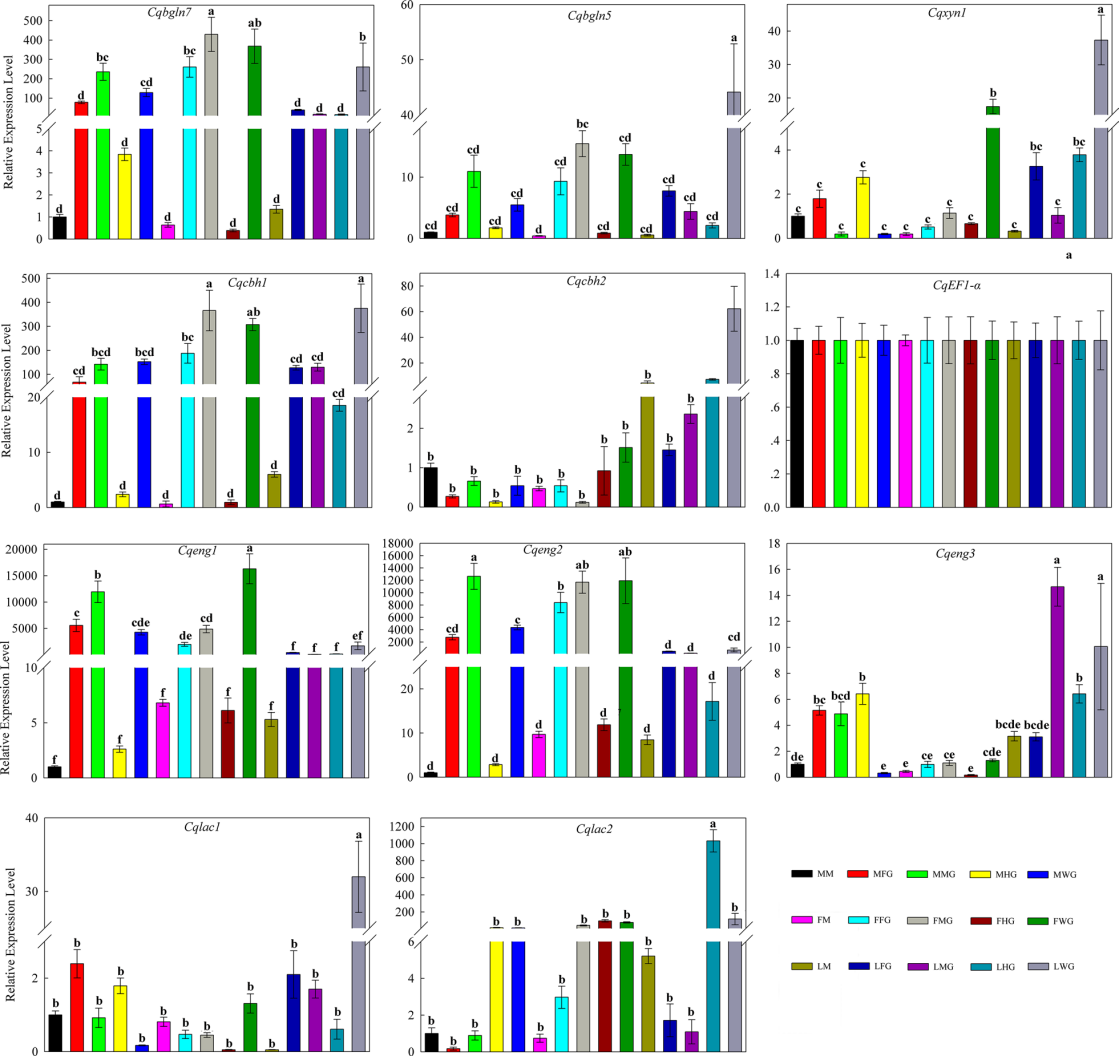
Quantitative RT-PCR analysis of expression 10 candidate genes in different intestinal sections of *C. buqueti*

To help elucidate the expression patterns of lignocellulolytic enzyme-encoding genes in the gut of *C. buqueti*, qRT-PCR was conducted on 10 such genes, namely three endoglucanase genes (*Cqeng1*, *Cqeng2* and *Cqeng3*), two β-glucosidase genes (*Cqbgln5* and *Cqbgln7*), two exoglucanase genes (*Cqcbh1* and *Cqcbh2*), one xylanase gene (*Cqxyn1*) and two Lac genes (*Cqlac1* and *Cqlac2*), with the *EF1*-*ɑ* gene acting as the reference gene, using the primers listed in Table 4. Expression of these 10 genes was detected in the mouthparts, foregut, midgut, hindgut and whole gut of adult females, adult males and larvae. Seven cellulase genes were mainly expressed in the foregut and midgut, with higher expression levels in the midgut. Expression patterns of the two β-glucosidase genes *Cqbgln5* and *Cqbgln7* were midgut > foregut > hindgut > mouthparts and midgut > foregut > mouthparts > hindgut, respectively. The *Cqbgln7* β-glucosidase gene was highly expressed in the intestines of both adults and larvae, with the highest expression level occurring in the midgut, which was higher than that of the other β-glucosidase gene *Cqbgln5*. Of the three endoglucanase genes, *Cqeng1* and *Cqeng2* showed high expression in all samples whereas the expression of *Cqeng3* was far lower but peaked in the midgut. The exoglucanase gene *Cqcbh1* was more highly expressed in all samples than the other exoglucanase gene *Cqcbh2*. The xylanase gene *Cqxyn1* exhibited low expression levels in all samples, except for the gut of larvae. The two Lac genes *Cqlac1* and *Cqlac2* were mainly expressed in the hindgut and their expression was higher in larvae than in adults (Fig. 7).

These findings show that cellulase genes were expressed at the highest level in the midgut of *C. buqueti*, with highest expression in endoglucanase genes followed by the β-glucosidase and exoglucanase genes, with expression being higher in adults than in larvae. Lignin degradation genes were expressed at the highest level in the hindgut, with higher expression levels in larvae than in adults. These findings indicate that the expression of endoglucanase and β-glucosidase genes primarily occurred in the adult midgut, whereas that of Lac primarily occurred in the hindgut of larvae. These results are in accord with those of the enzyme activity assays. Moreover, correlation analyses between enzyme activity data and qRT-PCR data were performed, which revealed that only the expression pattern of *Cqeng2* was significantly correlated to enzyme activity (Additional file 4: Table S4; Additional file 7: Table S5).

## Discussion

### High lignocellulase enzyme activities in the gut of *C. buqueti*

Cellulose degradation is generally attributed to the synergistic action of three classes of glycoside hydrolases: (1) endoglucanases, (2) exoglucanases and (3) β-glucosidases [44]. Endoglucanases have an open active site to bind to and cleave a cellulose molecule at any accessible point along the chain. In contrast, exocellulases are characterised by an active site inside a tunnel and bind only at one end of the cellulose chain. Lastly, cellobiases/β-glucosidases hydrolyse fragments are generated by exocellulases and generate individual monosaccharides [45, 46]. In this study, we used the 1,3-dinitrosalicylic acid (DNS) assay method to determine cellulase activities in the intestines of adult females, adult males and larvae. In adult females, endoglucanase activity was highest in the midgut and foregut, followed by the mouthparts and the hindgut. In males, endoglucanase activity was also highest in the midgut and foregut and was comparable to activity in larvae. CB activity was highest in the hindgut of adult females, in the hindgut and midgut of adult males and in the foregut and midgut of larvae. EG activity was highest in the midgut of adult females, in the mouthparts, foregut and midgut of adult males and in the midgut of larvae.

Insect cellulase was identified in the intestines of termites and cockroaches [47, 48] and subsequently detected in other insects [49–59]. Endogenous cellulase activity exists in insects and at least seven orders, comprising 28 species, have been found to contain a cellulase gene [6, 60]. Jiang [61] compared cellulase activities among three species belonging to different subfamilies of Cerambycidae. Duan [62] compared cellulase activity between *Monochamus alternatus* and *Cipangopaludina chinensis*, whereas Shi [63] compared xylanase and cellulase activities between three orders and three species of insects, including one member of Cerambycidae. Oppert [7] investigated cellulase activities in 68 species from eight orders of phytophagous insects, whereas Su [64] studied intestinal cellulase activities in 54 species of seven insect orders. Li [65] determined cellulase activities in 15 beetles. Taken together, these studies demonstrate that cellulase activity is limited by many factors, including substrate concentration and reaction time, and that results depend on the protein quantification methods used and other factors. Hence, it is difficult to directly compare the results from the current study with those from other reports [7]. Therefore, the purpose of this study was to determine the activities of lignocellulase enzymes in *C. buqueti* and to compare the activities of the different lignocellulolytic enzymes at different developmental stages and in different intestinal regions within *C. buqueti*.

We also determined activities of individual lignin degradation enzymes, such as Lac, LiP and MnP. Lac and MnP activities were highest in larvae, particularly in the midgut and hindgut. Lac was mainly distributed in the midgut of adult females, the hindgut of adult males and the midgut of the larvae. MnP activity was highest in the hindgut of adult females, mouthparts of adult males and hindgut and midgut of larvae. LiP was mainly distributed in the midgut of adult females, hindgut of adult males and midgut of larvae. Ander and Eriksson [66] noted that although Lac could efficiently degrade lignin, LiP had higher catalytic oxidoreduction potential and could catalyse a range of lignin compounds, including phenol, aromatic ethers, methoxy benzene, methyl alcohol and polycyclic aromatic compounds [67, 68]. The mechanism of action of MnP was similar to that of LiP [69]. These results indicate that *C. buqueti* has the ability to biodegrade lignin and cellulose, and that characterisation of its degradation system will be useful for using bamboo lignocellulose to produce biofuels.

### CAZyme families and their function in lignocellulose degradation in *C. buqueti*

CAZymes can be divided into six main categories: GHs, GTs, PLs, CEs, AAs and CBMs. According to the classification and definition of CAZyme genes [36], the functions of GHs, CEs, PLs and CBMs mainly relate to lignocellulosic polysaccharide degradation, whereas AAs play a more important role in the depolymerisation of lignin [38]. Zhang et al. [70] constructed an expressed sequence tag (EST) library of ergates, dinergates, nymphs, male and female termites and obtained 25,939 genes from which 509 CAZymes were identified, covering host and symbiotic cellulases. Poulsen et al. [71] sequenced the genome of the higher termite *Macrotermes natalensis* and obtained many CAZyme genes, including 30,297 GHs, 11,622 GTs, 4380 CBMs, 2729 CEs and 793 PLs. Mckenna [72] annotated 86 GH family genes in the *Anoplophora glabripennis* genome, including 57 GH1, 11 GH9, 2 GH5, GH45, GH48 and GH28 genes.

Most reported GHs are from microbes [36, 73–76] and invertebrates [77, 78]. In the *C. buqueti* transcriptome, 55 GH family genes were detected, including GH1, GH5, GHF7, GH9, GH45, GH48 and GH16 (Table 2). Eleven GH1 genes were found, most of which were putative β-glucosidases that can probably convert cellobiose and other oligosaccharides released from plant cell walls into monosaccharides. GH1 enzymes have broad catalytic and substrate specificities and function as β-xylosidases, β-glucuronidases, β-galactosidases, β-mannosidases or exo-β-1,4-glucanases, serving to hydrolyse substrates released from the hemicellulose matrix. CAZymes include > 40 GH45 cellulases and 4230 records of GH5, including the activities of enzymes like β-mannosidase (EC 3.2.1.25), chitosanase (EC 3.2.1.132), endo-β-1,4-glucanase (endocellulase, EC 3.2.1.4) and others [79]. Several Lamiinae longhorn beetles contain relatively large numbers of copies of GH5 cellulase [80, 81].

Several enzymes, including xylanases and esterases, are needed to completely degrade hemicellulose polysaccharides [82]. Only six typical xylanase genes were identified, but it was noted that 22 β-galactosidases, 25 mannosidases, 17 xyloglucosyltransferases, 82 arylesterases and 75 acetyl xylan esterases were present in the developmental transcriptome. *C. buqueti* α-mannosidase genes consisted of GH38 (EC 3.2.1.24) and GH47 families (EC 3.2.1.113). GH16 is another group of enzymes with xyloglucan: xyloglucosyltransferase activities and it encodes β-1,3-glucanases [83]. GH16 proteins have evolved to exhibit β-1,3-glucanase activity without a GNBP non-catalytic binding domain, and phylogenetic analysis suggests that the beta-1,3-glucanase function evolutionarily preceded its immune role [84–86]. Members of CE10 exhibit both carboxylesterase and xylanase activities [37], whereas AAs largely contribute to lignin breakdown [38]. A total of 131 AA proteins were identified from the developmental transcriptome of *C. buqueti*, including 10 AA1, 5 AA2, 46 GMC oxidoreductases (AA3 and AA4), four AA6 and other AAs (AA5, AA7 and AA8). The GMC family oxidoreductase is thought to supply hydrogen peroxide for LiP and MnP to participate in lignin degradation [39, 40]. Increasing reports on multicopper oxidases have demonstrated Lac activities [87, 88]. These findings suggest that insects have lignocellulose degradation-related enzymes and genes and that they differ across developmental stages and in different tissues.

In this study, biochemical techniques were used to demonstrate that lignocellulolytic enzyme activities in *C. buqueti* are highest in the midgut. These highly efficient enzymes could be introduced into microbes by synthetic biology to increase the yield of cellulase. Efficient lignocellulose degradation mechanisms by termites and other natural systems have provided important information on how lignocellulose can be exploited. By combining physical and chemical treatments with a natural enzyme system, it will be possible to achieve efficient hydrolysis of all carbohydrates in biomass under normal temperatures and pressure [89]. Sun [90] simulated a termite biotransformation system using properly comminuted biomass, adding specific glycosylhydrolases and lignin oxidase and separating aerobic and anaerobic reaction zones to achieve efficient lignocellulosic biomass biodegradation [90]. By contrast, research into the bamboo lignocellulose degradation mechanism in *C. buqueti* digestive is still in its infancy. Many significant biological problems must be resolved before rapid and effective bamboo lignocellulose degradation can be achieved, such as the mechanisms and biological functions of intestinal symbiotic bacteria.

## Conclusions

*Cyrtotrachelus buqueti*, a bamboo shoot snout beetle, is considered a pest by the bamboo industry. Using transcriptome analysis to dissect the mode-of-action of lignocellulose degradation in *C. buqueti,* this work provides a theoretical basis for the development of bamboo as a bioresource for the biofuel and bioenergy industries. Because larvae and adults feed mainly on bamboo shoots containing abundant lignocellulose, we hypothesised that *C. buqueti* utilise bamboo lignocellulose for development and growth. WGCNA was used to analyse the diversity of lignocellulose degradation enzymes, including CAZymes, during *C. buqueti* development. The results showed that CAZymes genes in the MEyellow module in larval and adult stages (when bamboo feeding takes place), rather than in egg and pupal stages, were consistent with the eating habits of *C. buqueti*. Of the three cellulases, enzyme activity assays showed that the activity of CBH was highest, followed by EG and CB, with the highest activity levels in the midgut. Of the three lignin degradation enzymes, Lac activity was highest and LiP activity was lowest, with activity being highest in the midgut. Gene expression results for different intestinal regions were consistent with enzyme activity assay results. Taken together, the findings revealed that *C. buqueti* has lignocellulose degradation-related enzymes and genes that are expressed differently according to the developmental stage, with the adult stage being associated with cellulose degradation and the larvae stage being associated with lignin degradation. In addition, different regions of the intestine had different functions, with the midgut being responsible for cellulose degradation and the hindgut for lignin degradation.

## Methods

### Insect collection

Larvae and adults of *C. buqueti* were collected in July 2017 from the bases of bamboo plants at a bamboo plantation in Muchuan City, Sichuan Province, China (N103°98′, E28°96′). All adults were used in the experiment 3 days after emergence [28]. Adults and larvae were reared in the laboratory at 25 °C ± 1 °C and 70% ± 10% relative humidity, with a 12 L:12 D photoperiod and fed a diet of bamboo shoots.

### Transcriptome data from *Cyrtotrachelus buqueti* Guérin-MéNeville and CAZyme family analysis

Transcriptomes from five different *C. buqueti* developmental stages, namely eggs, larvae, pupae, adult male and adult female, were used [35]. We downloaded raw data from the National Centre for Biotechnology Information (NCBI) (https://www.ncbi.nlm.nih.gov/) and focussed on genes associated with the lignocellulose degradation pathway. To identify genes involved in lignocellulose degradation, coding sequences were analysed using the dbCAN CAZyme annotation algorithm, which gives the hidden Markov model index files of various carbohydrate enzyme domains by hmmscan [91]. Weighted correlation network analysis (WGCNA) was used to analyse the 10,789 genes in the transcriptome [92].

### Reconstruction of a scale-free co-expression network using WGCNA

We used the co-expression network approach to reconstruct the scale-free co-expression network for *C. buqueti* and then built and mined the gene co-expression network. Using the WGCNA package [92], we first built a similarity matrix between all gene pairs using bi-weight mid-correlation based on normalised fragments per kilobase per million reads (FPKMs).

### Identification of functional modules

To identify functional modules in our reconstructed co-expression network, the adjacency matrix was further transformed to a topological overlap matrix using the WGCNA package. By setting the deepSplit parameter from 0 to 4 with the dynamic TreeCut package version 1.62, we found the optimal value to generate smaller clusters; a final deepSplit value of 4 was chosen and resulted in 19 modules (Fig. 2a). The relationship between modules was summarised by the eigenvalue ‘eigengene’, which represents the expression profile with weighted genes for each module [93].

### Pathway enrichment analysis and network analysis

We performed pathway enrichment analysis on the genes of interest, including enrichment in predefined pathways from the Kyoto Encyclopaedia of Genes and Genomes (KEGG) and Gene Ontology (GO) using the Cytoscape software platform (version 3.4) [94]. We used the degree of node metric to represent the number of connections for one node to the other nodes in the network and to identify the shortest path, represented by the fewest number of steps from one node to another [95].

### Assays of lignocellulolytic enzyme activity

A total of 165 female imagos, 165 male imagos and 165 larvae of *C. buqueti* were sampled to determine the activity of lignocellulolytic enzymes. First, the digestive system was dissected into mouthparts, foregut, midgut, hindgut, total intestine, and mouthparts + total intestine. Next, tissues were ground into 1 ml PH 5.6 PBS extraction buffer, the crude extract was centrifuged at 13,000× for 10 min at 4°C and the supernatant was collected. The supernatant represented the crude enzyme solution. Each replicate sample contained tissues from at least five insects and five biological replicates were conducted for each treatment.

The crude enzyme solution was used for the assays to determine lignocellulolytic enzyme activity. The assay method for endoglucanase (EC 3.2.1.4) and exoglucanase (EC 3.2.1.91) was performed as described by Ghose et al. [96], and β-glucosidase (EC 3.2.1.21) activity was assayed as described by Parry et al. [97]. Carboxymethyl cellulose (CMC), microcrystalline cellulose (MCC) and salicin were used as substrates for determination of endoglucanase, exoglucanase and β-glucosidase, respectively. First, 2 ml 1% CMC, MCC or salicin was added to a 25 ml test tube and preheated at 50 °C for 2–3 min. Second, 0.5 ml crude enzyme solution was added and incubated for 30 min at 50 °C. Next, 2.5 ml DNS was added and incubated for 5 min at 100 °C to immediately terminate the reaction. Finally, 25 ml PH 5.6 PBS was added and the optical density value was determined at a wavelength of 540 nm.

Lignin peroxidise (LiP)-like activity was measured according to the method by Shi et al. [98]. Briefly, veratryl alcohol (VA) was used as the substrate and the reaction was performed at PH 5.6 PBS. LiP activity was measured by monitoring the oxidation of VA at 310 nm. Laccase-like activity was measured according to the method used by Nakagawa et al. [99], in which 2, 2′-azino-bis (ABTS) was used as the substrate and enzyme activity was measured by monitoring oxidation of ABTS at 420 nm. Manganese peroxidise (MnP)-like activity was measured by monitoring oxidation of 2,6-dimethyl phenol (2,6-DMP) to coerulignone at 469 nm (ε469 = 49,600/mol cm) [98]. All assays were performed with five replicates.

### Tissue RNA extraction and qRT-PCR of lignocellulolytic enzyme genes in the *C. buqueti* digestive system

Thirty females, 30 males and 30 larvae that had been starved for 24 h were subjected to qRT-PCR assays. The five tissues (mouthparts, foregut, midgut, hindgut and intestine) of the three developmental stages (larvae, male and female) were rapidly extracted. The RNAprep Pure Tissue Kit (DP431; Tiangen Biotech, Beijing, China) was used to extract total RNA from the intestines of *C. buqueti*. All treatments used three biological replicates. The primers used in the qRT-PCR analysis are listed in Table 6. qRT-PCRs were conducted using the ABI StepOnePlus™ Real-Time PCR System. All qRT-PCRs were run using three biological replicates and analysed using the 2^−ΔΔCT^ method [100]; gene expression levels in other tissues were normalised to that in the mouthparts of male imagos, where the expression level of the genes in the mouthparts of males was set to one.

**Table 6:** The primer sequence of qRT-PCR

Gene ID	Gene name	Forward primer 5′ → 3′	Reverse primer 5′ → 3′
c31852_g1_i2	*Cqbgln7*	CAACATAGAGCCAGTCGTCACAAT	GCGTATTCAAGGAAAGCAGGGATT
c31732_g6_i2	*Cqbgln5*	TCCTGCTTTCGCTGACTATGC	CCATTCGTAACCACCTCCACAA
c20964_g1_i1	*Cqeng1*	GCTGCCAACATTGCGTTCATA	GACTCGGGTAAATCAGGACAAGAA
c31184_g1_i1	*Cqeng2*	GTCAAGCATCCACTACCAGATACT	TCCGCCACCATCACATCC
c63642_g1_i1	*Cqeng3*	CTTCAGGCATTGACGGTAACAC	GACAAGGAAACAAGCACAACACAT
c23242_g1_i1	*Cqcbh1*	CTGCTGCTACTGATTGGGCTATA	CGGTGGCTCTTCATAATTGTTGTT
c29519_g1_i1	*Cqcbh2*	CCATCAGCCAAAGGACCATCA	ACGGAATTGTGCCACGAGAA
c68284_g1_i1	*Cqxyn1*	CAACCGCTACCGCACCAT	GTCGCAGCACCTCCATGT
c27827_g1_i1	*Cqlac1*	CGGTGTCTACGGCAGCAT	CCAATCGGAGAGGAGGATAACG
c28149_g1_i1	*Cqlac2*	GGCGGTGCTGGAGTATGAG	TTGTGGAAGGTGTAACGGTTGT
EF1-α	*CqEF1*-*α*	AAGAATGGACAGACTCGTGAAC	AATGGAACAGCAGCAGGATT

### Statistical analysis

Statistical analyses were performed using SPSS 19.0 (IBM SPSS, Armonk, NY, USA). Descriptive data are expressed as mean ± standard error of mean (SEM). The Student’s *t* test was used to compare means from two groups. Intergroup comparisons involving more than two groups were performed using analysis of variance (ANOVA). A *p* value less than 0.05 indicated a statistically significant difference.

## Additional files

**Additional file 1: Table S1.** The CAYz family in the transcriptome. The family and numbers are listed.

**Additional file 2: Table S2.** The annotation of predicted CAZyme genes in the transcriptome.

**Additional file 3: Table S3.** Eigengene of each module in each sample.

**Additional file 4: Table S4.** Nutritional value per 100 g of bamboo shoots.

**Additional file 5: Figure S1.** Clustering heatmap of CAZyme gene expression. The expression patterns of GHs (a), GTs (b), CEs (c), CBMs (d) and AAs (e) in the MEyellow module. GHs: glycoside hydrolases, GTs: glycosyltransferases, CEs: carbohydrate esterases, CBMs: carbohydrate-binding domains, PLs: polysaccharide lyases, AAs: auxiliary activities and CAZyme: carbohydrate-active enzymes.

**Additional file 6: Figure S2.** Phylogenetic tree of cellulase genes. β-glucosidase genes (a), endoglucanase genes (b) and laccase genes (c) defined in the transcriptome of Lesham and Chishiu. The red line represents the branch of proteins in *C. buqueti.*

**Additional file 7: Table S5.** Correlation analysis between enzyme activity data and qRT-PCR data.

## Abbreviations

*C. buqueti*: *Cyrtotrachelus buqueti*
WGCNA: weighted gene co-expression network analysis
NBUS: natural biomass utilisation systems
RT-PCR: reverse transcription polymerase chain reaction
FG: foregut
MG: midgut
HG: hindgut
GHs: glycoside hydrolases
GTs: glycosyltransferases
CEs: carbohydrate esterases
CBMs: carbohydrate binding domains
PLs: polysaccharide lyases
AAs: auxiliary activities
CAZyme: carbohydrate-active enzymes
MnP: manganese peroxidases
LiP: lignin peroxidase
Lac: laccase
CBH: exoglucanase
EG: endoglucanase
CB: β-glucosidases
cDNA: complementary deoxyribonucleic acid
RNA: ribonucleic acid
NCBI: National Centre for Biotechnology Information
GO: gene ontology
KEGG: Database of Kyoto Encyclopaedia of Genes and Genomes
FPKM: fragments per kilobase of transcript, per million fragments sequenced
ABTS: [2,2′-Azino-bis(3-ethylbenzothiazoline-6-sulfonic acid)]

## References

[1] Claassen PM, Lier JV, Contreras AL, Niel EV, Sijtsma L, Stams AM (1999). Utilisation of biomass for the supply of energy carriers. Appl Microbiol Biotechnol.

[2] Demain AL, Newcomb M, Wu JH (2005). Cellulase, clostridia, and ethanol. Microbiol Mol Biol Rev.

[3] Himmel ME, Ding SY, Johnson DK, Adney WS, Nimlos MR, Brady JW (2007). Biomass recalcitrance: engineering plants and enzymes for biofuels production. Science.

[4] Fujita A, Hojo M, Aoyagi T, Hayashi Y, Arakawa G, Tokuda G (2010). Details of the digestive system in the midgut of *Coptotermes formosanus* Shiraki. J Wood Sci.

[5] Sun JZ, Scharf ME (2010). Exploring and integrating cellulolytic systems of insects to advance biofuel technology. Insect Sci.

[6] Watanabe H, Tokuda G (2010). Cellulolytic systems in insects. Annu Rev Entomol.

[7] Oppert C, Klingeman WE, Willis JD, Oppert B, Futentes HJL (2010). Prospecting for cellulolytic activity in insect digestive fluids. Comp Biochem Physiol B.

[8] Xie SX, Syrenne R, Sun S, Yuan JS (2014). Exploration of natural biomass utilization systems (NBUS) for advanced biofuel- from systems biology to synthetic design. Curr Opin Biotechnol.

[9] Shi WB, Xie SX, Chen XY, Sun S, Zhou X, Liu LT (2013). Comparative genomic analysis of the endosymbionts of herbivorous insects reveals ecoenvironmental adaptations: biotechnology applications. PLoS Genet.

[10] Sun JZ, Zhou XG, Liu TX, Kang L (2010). Utilization of lignocellulose-feeding insects for viable biofuels: an emerging and promising area of entomological science. Recent advances in entomological research.

[11] Martin MM (1983). Cellulose digestion in insects. Comp Biochem Physiol.

[12] Prins RA, Kreulen DA (1991). Comparative aspects of plant cell wall digestion in insects. Anim Feed Sci Technol.

[13] Itakura S, Ueshima K, Tanaka H, Enoki A (1995). Degradation of wood components by subterranean termite, *Coptoterme formosanus* Shiraki. J Jpn Wood Res Soc.

[14] Scurlock JO, Dayton DC, Hames B (2000). Bamboo: an overlooked biomass resource. Biomass Bioenergy.

[15] Isagi Y, Kawahara T, Kamo K (1993). Biomass and net production in a bamboo *Phyllostachys bambusoides* stand. Ecol Res.

[16] Liese W (1987). Research on bamboo. Wood Sci Technol.

[17] Yuan Z, Wen Y, Kapu NS, Beatson R, Mark MD (2017). A biorefinery scheme to fractionate bamboo into high-grade dissolving pulp and ethanol. Biotechnol Biofuels.

[18] Yuan Z, Wen Y, Kapu NS (2017). Ethanol production from bamboo using mild alkaline pre-extraction followed by alkaline hydrogen peroxide pretreatment. Bioresour Technol.

[19] Yuan Z, Wen Y (2017). Evaluation of an integrated process to fully utilize bamboo biomass during the production of bioethanol. Bioresour Technol.

[20] Wi SG, Lee DS, Nguyen QA, Bae HJ (2017). Evaluation of biomass quality in short-rotation bamboo (*Phyllostachys pubescens*) for bioenergy products. Biotechnol Biofuels.

[21] Littlewood J, Lei W, Turnbull C, Murphy RJ (2013). Techno-economic potential of bioethanol from bamboo in China. Biotechnol Biofuels.

[22] Xiao X, Bian J, Li MF, Xu H, Xiao B, Sun RC (2014). Enhanced enzymatic hydrolysis of bamboo (*Dendrocalamus giganteus* Munro) culm by hydrothermal pretreatment. Bioresour Technol.

[23] Sun SN, Cao XF, Zhang XM, Feng X, Sun RC, Jones GL (2014). Characteristics and enzymatic hydrolysis of cellulose-rich fractions from steam exploded and sequentially alkali delignified bamboo (*Phyllostachys pubescens*). Bioresour Technol.

[24] Yang Z, Zhang M, Xin D, Wang J, Zhang J (2014). Evaluation of aqueous ammonia pretreatment for enzymatic hydrolysis of different fractions of bamboo shoot and mature bamboo. Bioresour Technol.

[25] Liang C (2010). Research progress of the utilization of bamboo biomass energy. World Bamboo Rattan.

[26] Dingze M, Luo QH, Min S, Wei W (2012). Extraction and identification of cuticular semiochemical components of *Cyrtotrachelus buqueti* Guerin-Meneville (Coleoptera: Curculionidae). Acta Entomol Sin.

[27] Yang YJ, Wang SF, Gong JW, Liu C, Mu C, Qin H (2009). Relationships among *Cyrtotrachelus buqueti* larval density and wormhole number and bamboo shoot damage degree. Chin J Appl Ecol.

[28] Yang H, Yang W, Yang CP, Cai Y, Pu YF, Fu YW (2015). Mating behavior of *Cyrtotrachelus buqueti* (Coleoptera: Curculionidae). Acta Entomol Sin.

[29] Vigueras G, Paredeshernández D, Revah S, Valenzuela J, Olivareshernández R, Le BS (2017). Growth and enzymatic activity of *Leucoagaricus gongylophorus*, a mutualistic fungus isolated from the leaf-cutting ant *Atta mexicana*, on cellulose and lignocellulosic biomass. Lett Appl Microbiol.

[30] Su L, Yang L, Huang S, Li Y, Su X, Wang F (2017). Variation in the gut microbiota of termites (*Tsaitermes ampliceps*) against different diets. Appl Biochem Biotechnol.

[31] Bhatia R, Gallagher JA, Gomez LD, Bosch M (2017). Genetic engineering of grass cell wall polysaccharides for biorefining. Plant Biotechnol J.

[32] Auer L, Lazuka A, Sillamdussès D, Miambi E, O’donohue M, Hernandezraquet G (2017). Uncovering the potential of termite gut microbiome for lignocellulose bioconversion in anaerobic batch bioreactors. Front Microbiol.

[33] Wang H, Rehman KU, Liu X, Yang Q, Zheng L, Li W (2017). Insect biorefinery: a green approach for conversion of crop residues into biodiesel and protein. Biotechnol Biofuels.

[34] de Sousa G, Dos Santos VC, de Figueiredo Gontijo N, Constantino R, e Silva GD, Bahia AC, Gomes FM, de Alcantara Machado E (2017). Morphophysiological study of digestive system litter-feeding termite *Cornitermes cumulans* (Kollar, 1832). Cell Tissue Res.

[35] Yang H, Su T, Yang W, Yang C, Lin L, Chen Z (2017). The developmental transcriptome of the bamboo snout Beetle *Cyrtotrachelus buqueti* and insights into candidate pheromone-binding proteins. PLoS ONE.

[36] Lombard V, Ramulu HG, Drula E, Coutinho PM, Henrissat B (2014). The carbohydrate-active enzymes database (CAZy) in 2013. Nucleic Acids Res.

[37] Zhao Z, Liu H, Wang C, Xu JR (2014). Erratum to: comparative analysis of fungal genomes reveals different plant cell wall degrading capacity in fungi. BMC Genom.

[38] Levasseur A, Drula E, Lombard V, Coutinho PM, Henrissat B (2013). Expansion of the enzymatic repertoire of the CAZy database to integrate auxiliary redox enzymes. Biotechnol Biofuels.

[39] Prongjit M, Sucharitakul J, Palfey BA, Chaiyen P (2013). Oxidation mode of pyranose 2-oxidase is controlled by pH. Biochemistry.

[40] Zámocký M, Hallberg M, Ludwig R, Divne C, Haltrich D (2004). Ancestral gene fusion in cellobiose dehydrogenases reflects a specific evolution of GMC oxidoreductases in fungi. Gene.

[41] Baldrian P, Valaskova V (2008). Degradation of cellulose by basidiomycetous fungi. FEMS Microbiol Rev.

[42] Luo C, Liu A, Long W, Liao H, Yang Y (2017). Transcriptome analysis of *Cyrtotrachelus buqueti* in two cities in China. Gene.

[43] Yang YJ, Qin H, Deng GM, Wang SF, Liao LR, Liu C (2011). Larvae population dynamics of C*yrtatrachelus buqueti* and the forecasting models with climate factors. Scientia Silvae Sinicae.

[44] Clark AJ (1996). Biodegradation of cellulose: enzymology and biotechnology. Colourage.

[45] Holtzapple M, Cognata M, Shu Y, Hendrickson C (1990). Inhibition of *Trichoderma reesei* cellulase by sugars and solvents. Biotechnol Bioeng.

[46] Gruno M, Väljamäe P, Pettersson G, Johansson G (2004). Inhibition of the *Trichoderma reesei* cellulases by cellobiose is strongly dependent on the nature of the substrate. Biotechnol Bioeng.

[47] Cleveland LR (1924). The physiological and symbiotic relationships between the intestinal protozoa of termites and their host, with special reference to *Reticulitermes flavipes* kollar. Biol Bull.

[48] Cleveland LR, Hall SR, Sanders EP, Collier J (1934). The wood-feeding roach *Cryptocercus*, its protozoa, and symbiosis between protozoa and roach. Ann Entomol Soc Am.

[49] Watanabe H, Noda H, Tokuda G, Lo N (1998). A cellulase gene of termite origin. Nature.

[50] Kim N, Choo YM, Lee KS, Hong SJ, Seol KY, Je YH (2008). Molecular cloning and characterization of a glycoside hydrolase family 9 cellulase distributed throughout the digestive tract of the cricket *Teleogryllus emma*. Comp Biochem Physiol B.

[51] Zhang D, Lax AR, Raina AK, Bland JM (2009). Differential cellulolytic activity of native-form and C-terminal tagged-form cellulase derived from *Coptotermes formosanus* and expressed in *E. coli*. Insect Biochem Mol Biol.

[52] Ni JF, Takehara M, Watanabe H (2010). Identification of activity related amino acid mutations of a GH9 termite cellulase. Bioresour Technol.

[53] Shimada K, Maekawa K (2010). Changes in endogenous cellulase gene expression levels and reproductive characteristics of primary and secondary reproductives with colony development of the termite *Reticulitermes speratus* (Isoptera: Rhinotermitidae). J Insect Physiol.

[54] Adlakha N, Rajagopal R, Kumar S, Reddy VS, Yazdani SS (2011). Synthesis and characterization of chimeric proteins based on cellulase and xylanase from an insect gut bacterium. Appl Environ Microbiol.

[55] Sethi A, Kovaleva ES, Slack JM, Brown S, Buchman GW, Scharf ME (2013). A GHF7 cellulase from the protist symbiont community of *Reticulitermes flavipes* enables more efficient lignocellulose processing by host enzymes. Arch Insect Biochem Physiol.

[56] Shelomi M, Watanabe H, Arakawa G (2014). Endogenous cellulase enzymes in the stick insect (*Phasmatodea*) gut. J Insect Physiol.

[57] Mei HZ, Xia DG, Zhao QL, Zhang GZ, Qiu ZY, Qian P (2015). Molecular cloning and characterization of a novel cellulase gene (Bh-EGaseI) in the Beetle *Batocera horsfieldi*. Gene.

[58] Hatefi A, Makhdoumi A, Asoodeh A, Mirshamsi O (2017). Characterization of a bi-functional cellulase produced by a gut bacterial resident of Rosaceae branch borer Beetle, *Osphranteria coerulescens* (Coleoptera: Cerambycidae). Int J Biol Macromol.

[59] Willis JD, Grant JN, Mazarei M, Kline LM, Rempe CS, Collins AG (2017). The TcEG1 Beetle (*tribolium castaneum*) cellulase produced in transgenic switch grass is active at alkaline PH and auto-hydrolyzes biomass for increased cellobiose release. Biotechnol Biofuels.

[60] Calderóncortés N, Watanabe H, Canocamacho H, Zavalapáramo G, Quesada M (2010). CDNA cloning, homology modelling and evolutionary insights into novel endogenous cellulases of the borer Beetle *Oncideres albomarginata* chamela (cerambycidae). Insect Mol Biol.

[61] Jiang SN, Yin YP, Wang ZK (1996). Studies on the cellulases resource in some species of longicorn borers (Coleoptera: Cerambycidae). Scientia Silvae Sinicae.

[62] Duan X, Zhang JJ, Zhu J, Cai HH, Chen FF, Guo AW (2009). Comparison research on cellulase system from *Monochamus alternatus* and *Cipangopaludina chinensis*. J Yunnan Agric Univ.

[63] Shi WB, Ding SY, Yuan JS (2011). Comparison of insect gut cellulase and xylanase activity across different insect species with distinct food sources. BioEnergy Res.

[64] Su LJ, Zhang HF, Yin XM, Chen M, Wang FQ, Xie H (2013). Evaluation of cellulolytic activity in insect digestive fluids. Genet Mol Res.

[65] Li YL, Xue HJ, Hu CX, Yang XK (2013). Comparation of gut cellulase activity in FOUR herbivorous beetles. Scientia Silvae Sinicae.

[66] Ander P, Eriksson KE (1976). The importance of phenol oxidase activity in lignin degradation by the white-rot fungus *Sporotrichum pulverulentum*. Arch Microbiol.

[67] Kersten PJ, Kalyanaraman B, Hammel KE, Reinhammar B, Kirk TK (1990). Comparison of lignin peroxidase, horseradish peroxidase and laccase in the oxidation of methoxybenzenes. Biochem J.

[68] Massimo Bietti A, Steen Steenken C (1998). Lifetime, reduction potential and base-induced fragmentation of the veratryl alcohol radical cation in aqueous solution. Pulse radiolysis studies on a ligninase “mediator”. J Phys Chem A.

[69] Hofrichter M (2002). Review: lignin conversion by manganese peroxidase (MnP). Enzyme Microb Technol.

[70] Zhang D, Lax AR, Henrissat B, Coutinho P, Katiya N, Nierman WC (2012). Carbohydrate-active enzymes revealed in *Coptotermes formosanus* (Isoptera: Rhinotermitidae) transcriptome. Insect Mol Biol.

[71] Poulsen M, Hu H, Li C, Chen Z, Xu L, Otani S (2014). Complementary symbiont contributions to plant decomposition in a fungus-farming termite. Proc Natl Acad Sci USA.

[72] Mckenna DD, Scully ED, Pauchet Y, Hoover K, Kirsch R, Geib SM (2016). Genome of the Asian longhorned Beetle (*Anoplophora glabripennis*), a globally significant invasive species, reveals key functional and evolutionary innovations at the Beetle-plant interface. Genome Biol.

[73] Mba MF, Davies GJ, Drancourt M, Henrissat B (2012). Genome analyses highlight the different biological roles of cellulases. Nat Rev Microbiol.

[74] Berlemont R (2017). Distribution and diversity of enzymes for polysaccharide degradation in fungi. Sci Rep.

[75] Berlemont R, Martiny AC (2013). Phylogenetic distribution of potential cellulases in bacteria. Appl Environ Microbiol.

[76] Berlemont R, Martiny AC (2015). Genomic potential for polysaccharides deconstruction in bacteria. Appl Environ Microbiol.

[77] Guo R, Ding M, Zhang SL, Xu G, Zhao F (2008). Molecular cloning and characterization of two novel cellulase genes from the mollusc *Ampullaria crossean*. J Comp Physiol B.

[78] Rahman MM, Akira I, Takao O (2014). Characterization of a GHF45 cellulase, AkEG21, from the common sea hare *Aplysia kurodai*. Front Chem.

[79] Liu J, Song K, Teng H, Zhang B, Li W, Xue H (2015). Endogenous cellulolytic enzyme systems in the longhorn Beetle *Mesosa myops* (Insecta: Coleoptera) studied by transcriptomic analysis. Acta Biochim Biophys Sin.

[80] Pauchet Y, Kirsch R, Giraud S, Vogel H, Heckel DG (2014). Identification and characterization of plant cell wall degrading enzymes from three glycoside hydrolase families in the cerambycid Beetle *Apriona japonica*. Insect Biochem Mol Biol.

[81] Scully ED, Hoover K, Carlson JE, Tien M, Geib SM (2013). Midgut transcriptome profiling of *Anoplophora glabripennis*, a lignocellulose degrading cerambycid Beetle. BMC Genom.

[82] Sun J, Tian C, Diamond S, Glass NL (2012). Deciphering transcriptional regulatory mechanisms associated with hemicellulose degradation in *neurospora crassa*. Eukaryot Cell.

[83] Rai KM, Balasubramanian VK, Welker CM, Pang M, Mei MH, Mendu V (2015). Genome wide comprehensive analysis and web resource development on cell wall degrading enzymes from phyto-parasitic nematodes. BMC Plant Biol.

[84] Bragatto I, Genta FA, Ribeiro AF, Terra WR, Ferreira C (2010). Characterization of a β-1,3-glucanase active in the alkaline midgut of Spodoptera frugiperda larvae and its relation to β-glucan-binding proteins. Insect Biochem Mol Biol.

[85] Genta FA, Bragatto I, Terra WR, Ferreira C (2009). Purification, characterization and sequencing of the major beta-1,3-glucanase from the midgut of Tenebrio molitor larvae. Insect Biochem Mol Biol.

[86] Barbosa EG (2015). Multiple horizontally acquired genes from fungal and prokaryotic donors encode cellulolytic enzymes in the bdelloid rotifer *Adineta ricciae*. Gene.

[87] Majumdar S, Lukk T, Solbiati JO, Bauer S, Nair SK, Cronan JE (2014). Roles of small laccases from *Streptomyces* in lignin degradation. Biochemistry.

[88] Ihssen J, Reiss R, Luchsinger R, Thöny-meyer L, Richter M (2014). Biochemical properties and yields of diverse bacterial laccase-like multicopper oxidases expressed in *Escherichia coli*. Sci Rep.

[89] Wan Y, Qi B, Xing J, Sun JZ, Ding SY, Peterson JD (2013). What we can learn from natural biomass-utilization systems for developing novel bioreactors. Biological conversion of biomass for fuels and chemicals: explorations from natural utilization.

[90] Geng AL, Sun JZ, Xie RR, Wu J, Xiao N (2017). Natural bioconversion systems of lignocellulose, the processing characteristics and biomimetic applications. Biotechnol Bus.

[91] Yin Y, Mao X, Yang J, Chen X, Mao F, Xu Y (2012). dbCAN: a WEB resource for automated carbohydrate-active enzyme annotation. Nucleic Acids Res.

[92] Langfelder P, Horvath S (2008). WGCNA: an R package for weighted correlation network analysis. BMC Bioinform.

[93] Langfelder P, Horvath S (2007). Eigengene networks for studying the relationships between co-expression modules. BMC Syst Biol.

[94] Shannon P, Markiel A, Ozier O, Baliga NS, Wang JT, Ramage D (2003). Cytoscape: a software environment for integrated models of biomolecular interaction networks. Genome Res.

[95] Barabási AL, Oltvai ZN (2004). Network biology: understanding the cell’s functional organization. Nat Rev Genet.

[96] Ghose TK (2009). Measurement of cellulase activities. Pure Appl Chem.

[97] Perry JD, Morris KA, James AL, Oliver M, Gould FK (2007). Evaluation of novel chromogenic substrates for the detection of bacterial beta-glucosidase. J Appl Microbiol.

[98] Yan S, Chai L, Tang C, Yang Z, Zhang H, Chen R (2013). Characterization and genomic analysis of kraft lignin biodegradation by the beta-proteobacterium *Cupriavidus basilensis* B-8. Biotechnol Biofuels.

[99] Nakagawa Y, Sakamoto Y, Kikuchi S, Sato T, Yano A (2010). A chimeric laccase with hybrid properties of the parental *Lentinula edodes* laccases. Microbiol Res.

[100] Kenneth JL, Schmittgen TD (2001). Analysis of relative gene expression data using real-time quantitative PCR and the 2^−ΔΔCT^ method. Methods.

