# Supplementary figures and images for: De novo transcriptome assembly of the bamboo snout beetle Cyrtotrachelus buqueti reveals ability to degrade lignocellulose of bamboo feedstock
Source: Biotechnol Biofuels. 2018 Oct 27;11:292. doi: 10.1186/s13068-018-1291-9 (PMC6204003; doi:10.1186/s13068-018-1291-9)

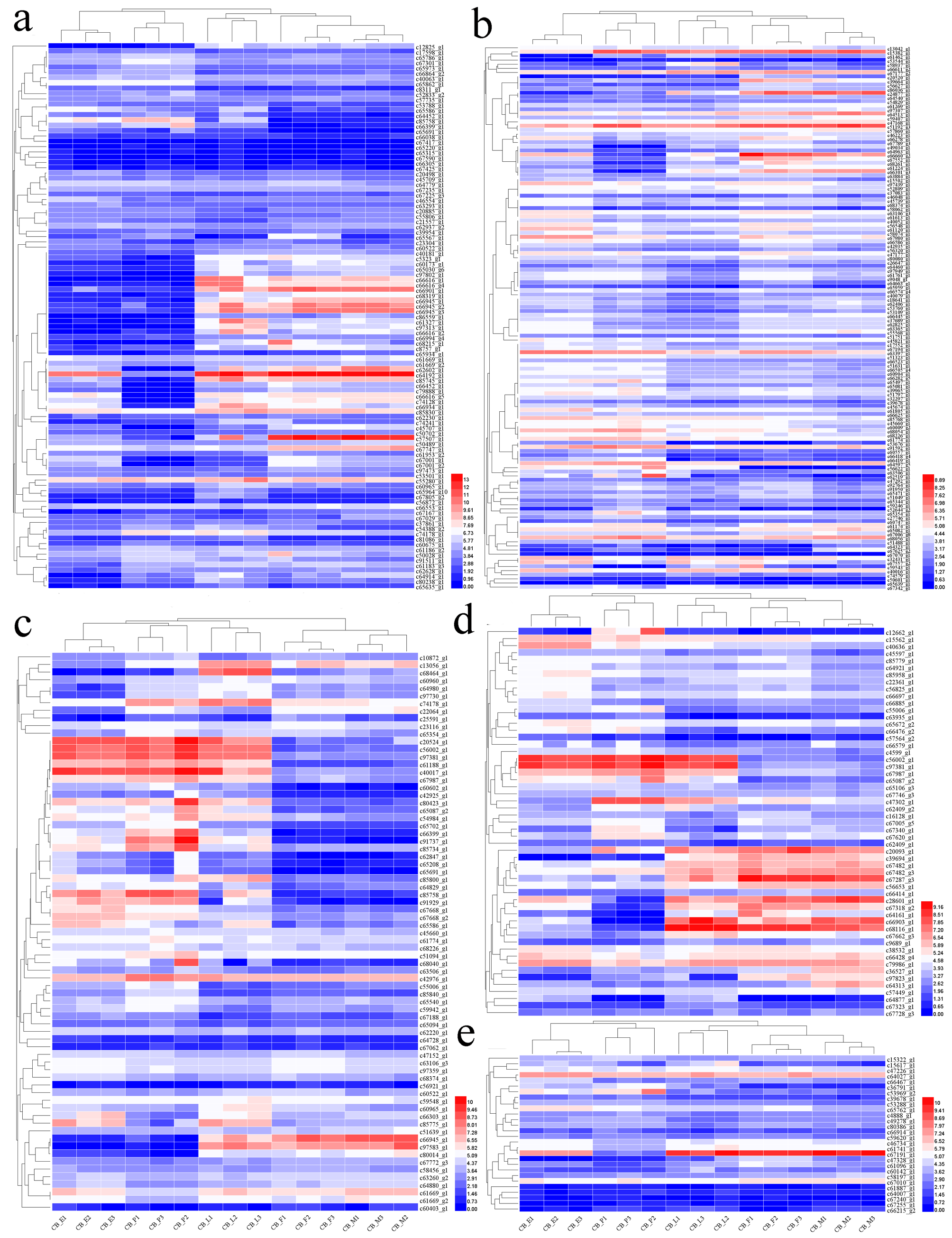

Supplement: Supplementary file 5 — Additional file 5: Figure S1. Clustering heatmap of CAZyme gene expression. The expression patterns of GHs (a), GTs (b), CEs (c), CBMs (d) and AAs (e) in the MEyellow module. GHs: glycoside hydrolases, GTs: glycosyltransferases, CEs: carbohydrate esterases, CBMs: carbohydrate-binding domains, PLs: polysaccharide lyases, AAs: auxiliary activities and CAZyme: carbohydrate-active enzymes. [file 13068_2018_1291_MOESM5_ESM.tif]

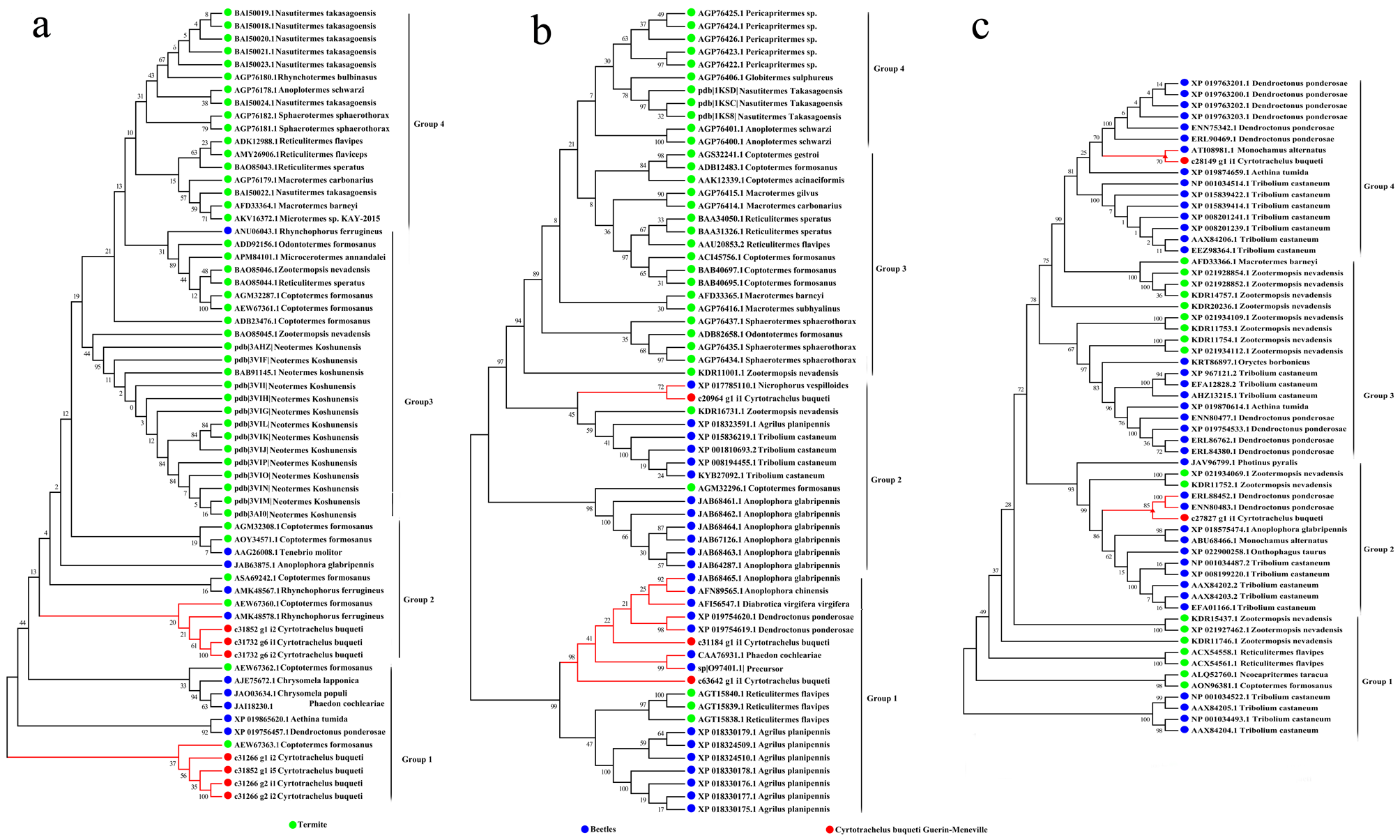

Supplement: Supplementary file 6 — Additional file 6: Figure S2. Phylogenetic tree of cellulase genes. β-glucosidase genes (a), endoglucanase genes (b) and laccase genes (c) defined in the transcriptome of Lesham and Chishiu. The red line represents the branch of proteins in C. buqueti. [file 13068_2018_1291_MOESM6_ESM.tif]
